# Reliability and performance modeling of an *M*^[*X*]^/*G*(*a*,*b*)/1 retrial queue with re-service and Balking behavior under working vacation policy

**DOI:** 10.1371/journal.pone.0354900

**Published:** 2026-08-06

**Authors:** Sundarapandiyan S, Nandhini S

**Affiliations:** Department of Mathematics, School of Advanced Sciences, Vellore Institute of Technology, Vellore, Tamil Nadu, India; City University of Hong Kong, HONG KONG

## Abstract

This study investigates a bulk arrival retrial queueing model characterized by two-phase service and a working vacation policy. Customers arrive in batches according to a Poisson process. If the server is unavailable, some customers may balk, while the remaining customers join the orbit and retry for service after a random period of time. The server provides a required first service phase followed by a second service phase; during this regular service period, customers who are unsatisfied or have specific needs may return for re-service. When the orbit is empty, the active server takes a working vacation, providing service to customers at a slower rate. This mechanism improves performance by reducing waiting times during low-traffic periods. The model employs supplementary variable techniques to determine system attributes, including probability generating functions for the mean queue length and mean orbit size. Numerical examples illustrate the impact of balking, vacation, and phase-type service on significant metrics.

## 1 Introduction

Queueing theory provides a fundamental component of performance analysis in various fields, including networks for computers, telephone service, production, medical care, and service administration. Fundamentally, it provides a mathematical framework for modeling congestion, delays, and resource utilization in systems characterized by competition for service among entities. Classical models, including the single-server Markovian and non-Markovian queues, have established a foundational framework for analyzing essential waiting line structure. The increasing number of components of real-world systems has revealed the shortcomings of conventional queueing models, especially in scenarios that include retrials, multiple service stages, server unreliability, working vacation, and customer impatience. This has prompted researchers to expand classical frameworks to include realistic features, resulting in the examination of another trial queue, two-phase service systems, re-service policies, balking behavior, and breakdown-repair dynamics.

A retrial queueing system occurs when blocked customers, who have no access to immediate service, remain permanently in the system but instead enter a virtual orbit, from which they attempt to receive service again after a random time frame. The retrial phenomenon is fundamental to contemporary communication and computing devices, where rejected calls, transmissions of data, or customer service inquiries automatically attempt to be processed again until successful completion occurs. The theoretical foundations of retrial queues were established in early surveys and bibliographies that compile methodologies, results, and unresolved challenges [1,2]. Classical studies offered detailed analyses of retrial phenomena, emphasizing both Markovian and non-Markovian frameworks, as well as their mathematical complexities [3]. An unreliable retrial queue with delayed repair under a modified vacation policy has been analyzed with an emphasis on performance prediction and intelligent computing using Adaptive Neuro-Fuzzy Inference Systems [4]. In addition, Atencia et al. [5] investigated a non-Markovian retrial queueing system with general retrial and service time distributions. Recent findings have been reported on retrial queues that incorporate feedback, delayed repair, and multiple types of customers [6]. Repeated queueing systems have been developed in the sustainable electric vehicle industry to improve the evaluation of green energy [7].

A single service phase is often insufficient to represent reliable service systems. Researchers have developed two-phase and multi-phase mechanisms for service, wherein customers experience a series of service stages prior to departure. Research has examined unreliable two-phase retrial models, along with the general service time distribution, working vacation, and the influence of initial failures on system performance [8]. Two-phase tandem retrial systems have been proposed to improve the quality of service in Internet-based applications [9]. Recent investigations in queueing theory have investigated advanced models, including the non-Markovian double retrial feedback model featuring optional services and repair under the working vacation policy [10]. Batch input recurrent queues with both passive and active breakdowns were investigated [11]. Various control mechanisms exist in unreliable dual-phase bulk systems that incorporate Bernoulli feedback and vacation [12]. The group arrival retrial queuing model includes two-phase services, feedback, and admission [13]. Recent research on optional re-service policies has considered batch arrivals, vacation policies, and unreliable servers, with emphasis on metaheuristic optimization for the balance of cost and system performance [14,15]. Optional re-service policies allow customers to receive additional service after completing the regular service, depending on system requirements. Such models have been widely studied in retrial queueing systems under various operating conditions, including working vacation, server breakdowns, and state-dependent services [16–18]. The advancements highlight that multi-phase service structures and re-service policies offer a more precise and adaptable framework for the analysis of complex service systems.

Modern queueing systems often assume that servers alternate between fully active and completely idle states. However, in many real-world situations, servers continue to provide service during vacation periods, but at a reduced service rate. The concept of the working vacation policy was first introduced by [19] in the early 2000s. Working vacation models have received significant attention because of their practical applications in telephone networks, call centers, and computer systems. For example, the model studied in [20] considers a general independent arrival process, exponential service times, a finite buffer, and a working vacation policy. Later studies extended this concept to retrial queues with unreliable servers, balking customers, and service interruptions [21,22]. Recent developments also include multi-server queueing systems with two-way communication [23]. In addition, cost analysis of queueing systems with working vacations, customer impatience, and optional second-phase service has also been investigated [24]. Several survey papers further highlight the wide range of applications of working vacation models in different industries [25]. These studies demonstrate both the theoretical importance and practical usefulness of working vacation policies in modern queueing systems.

In many service systems, customers may decide not to join the queue if they find it too long, which is known as balking. In some situations, customers may enter the system but leave before receiving service due to excessive waiting, a behavior called reneging. These customer behaviors are important in practical queueing systems because they directly affect system performance and customer satisfaction. Several researchers have studied retrial queueing models with balking, feedback mechanisms, optional service, and Bernoulli vacations [26]. Later, these models were extended to include unreliable servers and vacation interruptions in the *M*^[*X*]^/*G*/1 retrial feedback queue with balking and working vacation [27]. A single-server retrial queue with recurrent customers and balking under extended Bernoulli vacations was also analyzed to study non-exponential service behavior [28]. In addition, balking has been widely investigated along with feedback mechanisms, optional services, and bulk arrivals in non-Markovian retrial queueing systems [29,30]. In the present study, the balking probability is assumed to be fixed for analytical simplicity. However, in real-world systems, customer decisions may depend on factors such as congestion level, waiting time, and pricing policies. Therefore, incorporating state-dependent or adaptive balking behavior would make the model more realistic and represent an important direction for future research.

One of the important features of the realistic queueing systems is the presence of breakdowns and repair operations for the server. In practice, service systems may face unexpected failures of machines, processors, or servers, which may have a substantial impact on the system’s functioning. Hence, the addition of breakdown and repair processes makes queueing models more realistic and applicable in practice. Early research focused on the retrial queueing system with server breakdowns, exploring various working vacation rules [31]. The models were expanded subsequently to include multi-breakdowns, delayed repair, and imperfect coverage techniques [32–34]. Non-Markovian retrial queues with reneging, delayed repair, and working vacations under a server breakdown scenario have been studied in recent works [35].

Motivated by these developments, the present study proposes an integrated queueing model that simultaneously incorporates retrial phenomena, bulk arrivals, two-phase service processes, optional re-service mechanisms, working vacation policies, customer balking behavior, and breakdown–repair dynamics. These features correspond to practical operational phases, such as first-phase service (FPS), second-phase service (SPS), re-service, and reduced-efficiency service during working vacations.

The novelty of the present work lies in the unified integration of bulk arrivals, balking, retrial behavior, two-phase service, optional re-service, working vacations, and server breakdowns within a single analytical framework. Although many of these features have been extensively studied individually, their combined effects have received limited attention in the existing literature. The proposed model integrates these system features to better represent real-world service and industrial systems.

Earlier literature has studied retrial queueing models that incorporate working vacations and server failures. The present model, however, differs in several crucial respects. First, the presence of bulk arrivals significantly changes the arrival dynamics. Secondly, the implementation of two-phase service with the option of re-service increases the complexity of the service process. Third, the combined effects of balking, retry behavior, working vacations, and server breakdowns in both normal service and working vacation periods have not been studied together in the existing literature.

The remainder of this paper is organized as follows. Section 2 describes the proposed queueing model. Section 3 presents the steady-state probabilities of the system. Section 4 discusses the system performance measures. Section 5 examines several special cases, while Section 6 presents the cost optimization analysis. Section 7 presents the numerical analysis. Finally, Section 8 concludes the paper.

## 2 Characterization of the model

**Fig 1:**
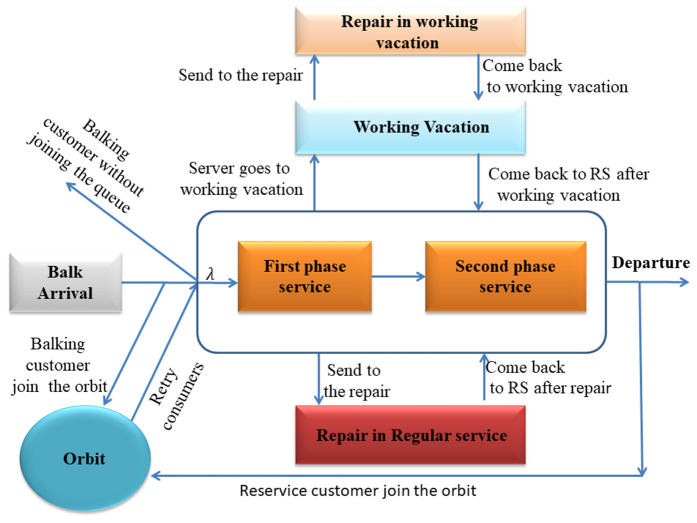
The model of bulk arrival retrial queue with two-phase service, re-service, balking customer and working vacation.

In this part, we introduce the bulk arrival retrial queue, which incorporates working vacation, two-phase service, balking customers, and a re-service policy. The description of our model is provided below, and the structural diagram is given in Fig 1:

- **Customer Arrival Rule:** Customers join the queue in the regular service period at an arrival rate of $\hat{\lambda }$ and in the working vacation (WV) period at an arrival rate of ${\hat{\lambda }}_{v}$, according to a Poisson process. For each $k=1,2,3,...,X_{k}$ has a common distribution representing the number of customers in the $K^{th}$ arrival batch. *X*(*z*) represents the probability generating function of *X*, and $P_{r}[X_{k}=n]=\phi_{n}$, *n* = 1, 2, 3, .... In this context, $X_{k}$ refers to the $K^{th}$ factorial moment of *X*(*z*).
- **Customer Balking:** Upon arrival, a customer may decide not to enter the system if it appears congested. This behavior is referred to as balking. In the proposed model, an arriving customer joins the orbit with probability $\overset{´}{b}$, while with probability $\overset{―}{b}=(1-\overset{´}{b})$, the customer balks and leaves immediately without receiving service. Similarly, the arriving customers join with probability ${\overset{´}{b}}_{v}$ and depart from the system with probability $\left(1-{\overset{´}{b}}_{v}\right)$ in the case of a working vacation.
- **Retrial Rule:** An arriving batch of customers receives service immediately if the server is available. If the server is busy, on working vacation, or under breakdown and repair, the blocked customers join the orbit and retry for service after a random period of time. Customers in the orbit make repeated retrial attempts according to the First In, First Out (FIFO) discipline. The retrial time follows the distribution function *A*(*t*), and its Laplace–Stieltjes transform (LST) is denoted by ${\hat{A}}^{*}(\hat{\upsilon })$.
- **Server Working Vacation Policy:** When the server finds that the orbit is empty, it enters a working vacation (WV) period. The vacation time is assumed to follow an exponential distribution with parameter *Q*. During the working vacation period, the server continues to provide service to arriving customers, but at a reduced service rate. If a customer joins the orbit while the server is in the working vacation state, the vacation is interrupted and the server immediately returns to the regular service mode. The working vacation service time distribution is denoted by ${\tilde{\tilde{M}}}_{v}(𝔱)$, and its Laplace–Stieltjes transform (LST) is represented by ${\tilde{\tilde{M}}}_{v}^{*}(\hat{\upsilon })$.
- **Regular Service Policy:** A single server provides service to customers according to a specified service discipline. The service process consists of two sequential phases, namely First Phase Service (FPS) and Second Phase Service (SPS). The customers complete their FPS with a probability of $\omega_{1}$, while they proceed to SPS with a probability of ω2. The service times of FPS and SPS follow general distributions represented by ${\tilde{\tilde{𝕄}}}_{1b}(𝔱)$ and ${\tilde{\tilde{𝕄}}}_{2b}(𝔱)$, respectively. Their Laplace–Stieltjes transforms (LSTs) are denoted by ${\tilde{\tilde{𝕄}}}_{1b}^{*}(\hat{\upsilon })$ and ${\tilde{\tilde{𝕄}}}_{2b}^{*}(\hat{\upsilon })$. Further, $E({\tilde{\tilde{𝕄}}}_{1b})$ and $E({\tilde{\tilde{𝕄}}}_{2b})$ denote the first moments of the FPS and SPS service times, respectively, while $E({\tilde{\tilde{𝕄}}}_{1b}^{2})$ and $E({\tilde{\tilde{𝕄}}}_{2b}^{2})$ represent the corresponding second moments.
- **Customer Re-Service Policy:** After completing service, a customer may not leave the system immediately. Instead, with probability p, the customer requires optional re-service for quality inspection, rework, or additional processing. With probability $\overset{―}{p}=(1-p)$, the customer leaves the system after completing the service. The re-service time is assumed to follow a general distribution with distribution function $\tilde{\tilde{𝕄}}r(𝔱)$. Its Laplace–Stieltjes transform (LST) is denoted by $\tilde{\tilde{𝕄}}r^{*}(\hat{\upsilon })$, while $E(\tilde{\tilde{𝕄}}r)$ and $E(\tilde{\tilde{𝕄}}r^{2})$ denote the first and second moments of the re-service time, respectively.
- **Server Failure Happens:** The server, while providing the First Phase Service (FPS), Second Phase Service (SPS), optional re-service, or service during a working vacation (WV), may experience an unexpected breakdown, resulting in service interruption. The times to server breakdown during the regular service states (FPS, SPS, and re-service) and the working vacation state are assumed to follow exponential distributions with rates δ and $\delta_{v}$, respectively.
- **The Server Repair Procedure:** The server maintenance begins immediately after a failure, regardless of whether it occurs during busy operational periods (FPS, SPS), re-service, or scheduled downtime. The server stops all operations until maintenance is completed. Customers getting service at the moment of the breakdown must await the restoration of service. During this downtime, the server remains inactive, awaiting repairs, a phase known as the server’s waiting time. The regular service and re-service timings are denoted by the functions $W_{ib}(𝔱),W_{r}(𝔱)$ and their Laplace-Stieltjes Transform (LST), $W_{ib}^{*}(y),W_{r}^{*}(y)$. The first two moments are denoted as ${𝔤}^{\left(if\right)},{𝔤}^{\left(is\right)}$ and ${𝔤}^{\left(ir\right)}$ for (*i* = 1,2). The server repair time during scheduled downtime is characterized by the distribution functions $G_{w}(𝔱)$, with their LST represented as $G_{w}^{*}(y)$, and the first two moments are $g_{v}^{1}$ and $g_{v}^{2}$, respectively.

**Notations**

The mathematical symbols and their meanings used in the proposed model are summarized in Table 1.

**Table 1:** List of notations.

Symbol	Description
$\hat{\lambda }$	Arrival rate of customers during the normal period
${\hat{\lambda }}_{v}$	Arrival rate during the working vacation period
*Q*	Rate parameter of working vacation duration
$\dot{a}$	Retrial rate of customers in the orbit
$\beta_{1b}$	Service rate in the first phase (FPS)
$\beta_{2b}$	Service rate in the second phase (SPS)
$\beta_{v}$	Service rate during working vacation
*p*	Probability of optional re-service
$\overset{´}{b}$	Probability that a customer joins the orbit
$\bar{b}=1-\overset{´}{b}$	Probability that a customer leaves the system without joining
${\overset{´}{b}}_{v}$	Probability that the arriving customers join in the orbit in WV period
δ	Server breakdown rate during the normal period
$\delta_{v}$	Server breakdown rate during working vacation
$\eta_{1}$	Repair rate after breakdown in the first phase
$\eta_{2}$	Repair rate after breakdown in the second phase
*U*_0_	Probability that the server is idle
$\pi_{q}$	Mean orbit size (average number of customers in orbit)
*T*	Probability of server unavailability during retrial
$\overset{ˇ}{x}$	Elapsed service/retrial time
*z*	Elapsed repair/vacation time
$\dot{a}(\hat{\upsilon })$	The hazard rate for retrial of $\hat{A}(\overset{ˇ}{x})$
$\beta_{ib}(\hat{\upsilon })$	The hazard rate for service of ${\tilde{\tilde{𝕄}}}_{ib}(\overset{ˇ}{x})$
$\beta_{v}(\hat{\upsilon })$	The hazard rate for slow rate service of ${\tilde{\tilde{M}}}_{v}(\overset{ˇ}{x})$
$\beta_{r}(x)$	The hazard rate for re-service of ${\tilde{\tilde{𝕄}}}_{r}(\overset{ˇ}{x})$
$\eta_{i}(z)$	The hazard rate for repair in two phase service of $W_{i}(z)$
$\eta_{r}(z)$	The hazard rate for repair in re-service of $W_{r}(z)$
φ(z)	The hazard rate for repair in working vacatiion of $G_{w}(z)$
$T_{n}(\overset{ˇ}{x},t)$	Probability that at time *t* there are *n* customers in the orbit,
	with elapsed retrial time in $\left(\overset{ˇ}{x},\overset{ˇ}{x}+d\overset{ˇ}{x}\right)$
${\tilde{S}}_{ib,n}(\overset{ˇ}{x},t)$	Probability that at time *t* there are *n* customers in the orbit,
	with elapsed normal two phase service time in $\left(\overset{ˇ}{x},\overset{ˇ}{x}+d\overset{ˇ}{x}\right)$
$\Omega_{ir,n}(\overset{ˇ}{x},t)$	Probability that at time *t* there are *n* customers in the orbit,
	with elapsed Re-service time in $\left(\overset{ˇ}{x},\overset{ˇ}{x}+d\overset{ˇ}{x}\right)$
${𝛷}_{v,n}(\overset{ˇ}{x},t)$	Probability that at time *t* there are *n* customers in the orbit,
	with elapsed working vacation service time in $\left(\overset{ˇ}{x},\overset{ˇ}{x}+d\overset{ˇ}{x}\right)$
${\overset{ˇ}{\overset{ˇ}{R}}}_{in}(\overset{ˇ}{x},z,t)$	Probability that at time *t* there are *n* customers in the orbit,
	with elapsed normal two phase service time υ and elapsed repair time *z*
${\overset{ˇ}{\overset{ˇ}{R}}}_{r}(\overset{ˇ}{x},z,t)$	Probability that at time *t* there are *n* customers in the orbit,
	with elapsed Re-service time $\overset{ˇ}{x}$ and elapsed repair time *z*
$F_{w,n}(\overset{ˇ}{x},z,t)$	Probability that at time *t* there are *n* customers in the orbit,
	with elapsed working vacation time $\overset{ˇ}{x}$ and elapsed repair time *z*

### 2.1 Application of the proposed model

In the shoe manufacturing industry, customer orders sometimes come in big quantities especially during the busy season or sales period. To process these bulk orders, several production stages such as cutting, stitching, and assembly operate simultaneously, which motivates the consideration of bulk arrivals. If a production station is busy, newly arriving batches may not receive immediate service and may retry later, which is represented through retrial behavior. Moreover, congestion or excessive waiting periods may lead to balking behaviour of certain customers who may decide not to join the system.

The shoe manufacturing process usually involves multiple sequential stages. For example, the first stage may include cutting and stitching, while the second stage involves assembly and finishing operations. This justifies the use of a two-phase service mechanism. After the production stage, a quality inspection may identify damaged products, which must be reworked or further processed. This is reflected in the optional re-service procedure.

In practical production environments, the system may continue operating at a reduced service rate during shift changes, maintenance activities, or reduced workforce availability. Such situations are effectively represented through the working vacation policy. At the same time, machine failures and interruptions are unavoidable in semi-automated and continuous production systems. During breakdown periods, production temporarily stops until repair is completed, after which the interrupted process resumes. Breakdowns may also occur during working vacation periods, further affecting system efficiency and increasing congestion.

Therefore, the simultaneous inclusion of bulk arrivals, balking, retrial behavior, two-phase service, optional re-service, working vacations, and server breakdowns within a unified framework is not merely a mathematical extension. Rather, it is essential for realistically representing modern manufacturing systems, where these operational factors occur simultaneously and jointly influence congestion, production efficiency, maintenance planning, and product quality.

## 3 Steady-state probabilities

We developed a mathematical equation to explain the structural difference differential equation in steady-state conditions. This equation included other factors, such as the duration of the retrial, FPS, SPS periods, re-service, WV, and repairs performed. We derive the governing equations for the state of the system using the SVT methodology. We have derived the PGF for the server states and the number of customers in both the orbit and the system.

Let us assume that $\hat{A}(0)=0,\;\hat{A}(\infty )=1,\;{\tilde{\tilde{𝕄}}}_{ib}(\overset{ˇ}{x})(0)=0,\;{\tilde{\tilde{𝕄}}}_{ib}(\overset{ˇ}{x})(\infty )=1,{\tilde{\tilde{𝕄}}}_{r}(\overset{ˇ}{x})(0)=0,{\tilde{\tilde{𝕄}}}_{r}(\overset{ˇ}{x})(\infty )=1,\;{\tilde{\tilde{M}}}_{v}(0)=0,\;{\tilde{\tilde{M}}}_{v}(\infty )=1,$$W_{r}(0)=0,W_{r}(\infty )=1,\;W_{ib}(0)=0,\;W_{ib}(\infty )=1\text{ and }G_{w}(0)=0,\;G_{w}(\infty )=1$ is continuous at *z* = 0. We postulate that the hazard rate operates as $\dot{a}(\overset{ˇ}{x}),\;\beta_{ib}(\overset{ˇ}{x}),\;\beta_{r}(\overset{ˇ}{x}),\;\beta_{v}(\overset{ˇ}{x}),\;\eta_{i}(z),\eta_{r}(z)\;and\;\varphi (z)\text{ where }(i=1,2)$ are evaluated in the sequence of retrial, regular service (FPS, SPS), lower rate service, breakdown and maintenance.


$$\begin{array}{ccc} & & \dot{a}(\overset{ˇ}{x})\;d(\overset{ˇ}{x})=\frac{d(\hat{A}(\overset{ˇ}{x}))}{\left(1-\hat{A}(\overset{ˇ}{x})\right)};\;\beta_{ib}(\overset{ˇ}{x})d\overset{ˇ}{x}=\frac{d({\tilde{\tilde{𝕄}}}_{ib}(\overset{ˇ}{x}))}{\left(1-{\tilde{\tilde{𝕄}}}_{ib}(\overset{ˇ}{x})\right)};\beta_{r}(\overset{ˇ}{x})d\overset{ˇ}{x}=\frac{d({\tilde{\tilde{𝕄}}}_{r}(\overset{ˇ}{x}))}{\left(1-{\tilde{\tilde{𝕄}}}_{r}(\overset{ˇ}{x})\right)};\\ & & \beta_{v}(\overset{ˇ}{x})d\overset{ˇ}{x}=\frac{d\;({\tilde{\tilde{M}}}_{v}(\overset{ˇ}{x}))}{\left(1-{\tilde{\tilde{M}}}_{v}(\overset{ˇ}{x})\right)};\;\eta_{i}(z)dz=\frac{d(W_{ib}(z))}{\left(1-W_{ib}(z)\right)};\eta_{r}(z)dz=\frac{d(W_{r}(z))}{\left(1-W_{r}(z)\right)};\\ & & \varphi (z)dz=\frac{d(G_{w}(z))}{\left(1-G_{w}(z)\right)},\text{where }(i=1,2). \end{array}$$

Define ${\hat{A}}^{0},{\tilde{\tilde{𝕄}}}_{ib}^{0},{\tilde{\tilde{𝕄}}}_{r}^{0},W_{r}^{0},{\tilde{\tilde{M}}}_{v}^{0},G_{w}^{0}$ as the hazard retrial, server busy, re-service, working vacation, and maintenance (FPS, SPS, and WV) times at 𝔱. We further assume the presence of a random variable,


$$\begin{array}{c} H(𝔱)=\left\{ \begin{array}{c} 0,\;\text{server is idle during the working vacation (WV),}\\ 1,\;\text{server is idle and available in regular service (FPS, SPS),}\\ 2,\;\text{server is providing regure service (FPS, SPS),}\\ 3,\;\text{server is providing re-service,}\\ 4,\;\text{server is busy in WV manner,}\\ 5,\;\text{server is undergoing repair WV period,}\\ 6,\;\text{server is undergoing repair FPS, SPS}\\ 7,\;\text{server is undergoing repair re-service.} \end{array}\right. \end{array}$$

The function H(𝔱) represents the server states, which are classified as (0, 1, 2, 3, 4, 5, 6, 7) depending on whether the server is idle, is engaged in the FPS, SPS, re-service, and WV periods, or is undergoing repair in the FPS, SPS, re-service, and WV periods. In addition, *N*(*t*) denotes the system size a*t* time t. {N(𝔱);H(𝔱);𝔱>0} constitutes a bivariate Markov process. Let H(𝔱) and *N*(*t*) be supplementary random variables defined as follows: If N≥0, then *H*(*t*) = 1. The server used *N*(*t*) to denote the elapsed retry duration. When N(t)≥0, H(t)=2, the server uses *N*(*t*) *t*o denote the elapsed service time during which it services customers using the regular service (FPS, SPS). When N(t)≥0,H(t)=3, i*t* follows *t*hat *N*(*t*) denotes the elapsed service time during which the server serves customers using re-service. When N(t)≥0,H(t)=4, it indicates tha*t N*(*t*) represents the elapsed service duration when customers are being attended to at a reduced service rate provided by the server. When N(t)≥0, H(t)=5,6, we refer to *N*(*t*) as the dura*t*ion of the repair time during server failure (WV, FPS, and SPS) and while under repair, respectively. When N(t)≥0, H(t)=7, we define *N*(*t*) as the duration of the repair time during server failure in the re-service period and while under repair.

**Ergodicity condition:**

We determine the conditions that are necessary and sufficient for the stability of the system. Theorem 2.1 explains the ergodicity requirement of the embedded Markov chain, especially during vacation or departure times, which leads to the conclusion of the chain. Let $\left(t_{n};n=1,2,...\right)$ represent the series of moments that arise after the completion of service or the end of vacation time. The collection of stochastic vectors *Xn* = (*H*(*tn*+); *N*(*tn*+)) constitutes a Markov chain, illustrating the embedded Markov chain relevant to our queueing system.

**Theorem 2.1.** *The Embedded Markov-Chain* $\left\{A_{𝔫};𝔫\in \hat{N}\right\}$ *is Ergodic if and only if* $\rho <{\hat{A}}^{*}(\hat{\lambda })$*.*

*Proof.* Let $\left\{A_{𝔫};𝔫\in \hat{N}\right\}$ constitutes an essential and aperiodic Markov-chain. We will be employing Foster’s Criterion to demonstrate ergodicity. An irreducible and aperiodic Markov chain is considered ergodic if there exists a non-negative function *k*(*i*) for *i* in $\hat{N}$ and ε>0, such that the mean drift $\phi_{i}=\overset{ˇ}{E}[k(A_{𝔫}+1)-k(A_{𝔫})/A_{𝔫}=i]<\infty$, ∀ $i\in \hat{N}$ and $\phi_{i}\leq -\varepsilon \;\forall \text{ i}\in \hat{N}$. We examine the function *k*(*i*)=*i*. Subsequently, we possess,


$$\begin{array}{c} \phi_{i}=\left\{ \begin{array}{c} \rho -1,\;i=0\\ \rho -\hat{A}(\hat{\lambda }),\;i=1,2,3,... \end{array}\right. \end{array}$$

The inequality $\rho <\hat{A}(\hat{\lambda })$ constitutes a sufficient condition for ergodicity. The identical inequality is also needed for ergodicity. According to Sennott et al. [36], the non-ergodicity of the Markov chain $\left\{A_{𝔫};𝔫\in \hat{N}\right\}$ may be assured if it meets Kaplan’s condition, which means that $\phi_{i}<\infty$ for every i≥0 and there exists a $i_{0}\in \hat{N}$ such that $\phi_{i}\geq 0$ for $i\geq i_{0}$. It is important to observe that Kaplan’s condition is fulfilled in our scenario, since $(r_{hi}=0$ for i<h−1 holds true for *h* > 0, where $R=(r_{hi})$ represents the sequence of transition matrix (single-step) $\left\{A_{𝔫};𝔫\in \hat{N}\right\}$. Consequently, $\rho \geq {\hat{A}}^{*}(\hat{\lambda })$ indicates the Markov chain exhibits non-ergodic behavior. □

### 3.1 The system’s steady-state solution

We use the supplementary variable approach to obtain the equations of the system presented below. The structure of this system determines its performance.


$$\begin{array}{ccc} & & ({\hat{\lambda }}_{v}+Q)U_{0}=QU_{0}+\left(\begin{array}{c} \int_{0}^{\infty }{\tilde{S}}_{ib,0}(\overset{ˇ}{x})\beta_{ib}(\overset{ˇ}{x})d\overset{ˇ}{x}+\int_{0}^{\infty }\Omega_{ir,0}(\overset{ˇ}{x})\beta_{r}(\overset{ˇ}{x})d\overset{ˇ}{x}\\ +\int_{0}^{\infty }{𝛷}_{v,0}(\overset{ˇ}{x})\beta_{v}(\overset{ˇ}{x})d\overset{ˇ}{x} \end{array}\right) \end{array}$$ (1)

$$\begin{array}{ccc} & & \frac{d}{d\overset{ˇ}{x}}T_{𝔫}(\overset{ˇ}{x})=-[\hat{\lambda }+\dot{a}(\overset{ˇ}{x})]T_{𝔫}(\overset{ˇ}{x}),𝔫\geq 1 \end{array}$$ (2)

$$\begin{array}{ccc} & & \frac{d}{d\overset{ˇ}{x}}{\tilde{S}}_{1b,0}(\overset{ˇ}{x})=-[\hat{\lambda }\overset{´}{b}+\beta_{1b}(\overset{ˇ}{x})]{\tilde{S}}_{1b,0}(\overset{ˇ}{x})+\hat{\lambda }(1-\overset{´}{b}){\tilde{S}}_{1b,0},𝔫=0 \end{array}$$ (3)

$$\begin{array}{ccc} & & \frac{d}{d\overset{ˇ}{x}}{\tilde{S}}_{1b,𝔫}(\overset{ˇ}{x})=-[\hat{\lambda }\overset{´}{b}+\beta_{1b}(\overset{ˇ}{x})]{\tilde{S}}_{1b,𝔫}(\overset{ˇ}{x})+\hat{\lambda }\overset{´}{b}\sum_{k=1}^{n}X_{k}{\tilde{S}}_{1b,n-k}(\overset{ˇ}{x})\\ & & \;\;\;+\int_{0}^{\infty }{\overset{ˇ}{\overset{ˇ}{R}}}_{1b,𝔫}(\overset{ˇ}{x},z)\eta_{1}(z)dz,𝔫\geq 1 \end{array}$$ (4)

$$\begin{array}{ccc} & & \frac{d}{d\overset{ˇ}{x}}{\tilde{S}}_{2b,0}(\overset{ˇ}{x})=-[\hat{\lambda }\overset{´}{b}+\beta_{2b}(\overset{ˇ}{x})]{\tilde{S}}_{2b,0}(\overset{ˇ}{x})+\hat{\lambda }(1-\overset{´}{b}){\tilde{S}}_{2b,0},𝔫=0 \end{array}$$ (5)

$$\begin{array}{ccc} & & \frac{d}{d\overset{ˇ}{x}}{\tilde{S}}_{2b,𝔫}(\overset{ˇ}{x})=-[\hat{\lambda }\overset{´}{b}+\beta_{2b}(\overset{ˇ}{x})]{\tilde{S}}_{2b,𝔫}(\overset{ˇ}{x})+\hat{\lambda }\overset{´}{b}\sum_{k=1}^{n}X_{k}{\tilde{S}}_{2b,n-k}(\overset{ˇ}{x})\\ & & \;\;\;+\int_{0}^{\infty }{\overset{ˇ}{\overset{ˇ}{R}}}_{2b,𝔫}(\overset{ˇ}{x},z)\eta_{2}(z)dz,𝔫\geq 1 \end{array}$$ (6)

$$\begin{array}{ccc} & & \frac{d}{d\overset{ˇ}{x}}\Omega_{1r,0}(\overset{ˇ}{x})=-[\hat{\lambda }\overset{´}{b}+\beta_{1r}(\overset{ˇ}{x})]\Omega_{1r,0}(\overset{ˇ}{x})+\hat{\lambda }(1-\overset{´}{b})\Omega_{1b,0},𝔫=0 \end{array}$$ (7)

$$\begin{array}{ccc} & & \frac{d}{d\overset{ˇ}{x}}\Omega_{1r,𝔫}(\overset{ˇ}{x})=-[\hat{\lambda }\overset{´}{b}+\beta_{1r}(\overset{ˇ}{x})]\Omega_{1r,𝔫}(\overset{ˇ}{x})+\hat{\lambda }\overset{´}{b}\sum_{k=1}^{n}X_{k}\Omega_{1r,n-k}(\overset{ˇ}{x})\\ & & \;\;\;+\int_{0}^{\infty }{\overset{ˇ}{\overset{ˇ}{R}}}_{1r,𝔫}(\overset{ˇ}{x},z)\eta_{r}(z)dz,𝔫\geq 1 \end{array}$$ (8)

$$\begin{array}{ccc} & & \frac{d}{d\overset{ˇ}{x}}\Omega_{2r,0}(\overset{ˇ}{x})=-[\hat{\lambda }\overset{´}{b}+\beta_{rb}(\overset{ˇ}{x})]\Omega_{2r,0}(\overset{ˇ}{x})+\hat{\lambda }(1-\overset{´}{b})\Omega_{2r,0},𝔫=0 \end{array}$$ (9)

$$\begin{array}{ccc} & & \frac{d}{d\overset{ˇ}{x}}\Omega_{2r,𝔫}(\overset{ˇ}{x})=-[\hat{\lambda }\overset{´}{b}+\beta_{2r}(\overset{ˇ}{x})]\Omega_{2r,𝔫}(\overset{ˇ}{x})+\hat{\lambda }\overset{´}{b}\sum_{k=1}^{n}X_{k}\Omega_{2r,n-k}(\overset{ˇ}{x})\\ & & \;\;\;+\int_{0}^{\infty }{\overset{ˇ}{\overset{ˇ}{R}}}_{2r,𝔫}(\overset{ˇ}{x},z)\eta_{r}(z)dz,𝔫\geq 1 \end{array}$$ (10)

$$\begin{array}{ccc} & & \frac{d}{d\overset{ˇ}{x}}{𝛷}_{v,0}(\overset{ˇ}{x})=-[{\hat{\lambda }}_{v}{\overset{´}{b}}_{v}+Q+\beta_{v}(\overset{ˇ}{x})]{𝛷}_{v,0}(\overset{ˇ}{x})+\hat{\lambda }(1-\overset{´}{b}){𝛷}_{v,0},𝔫=0 \end{array}$$ (11)

$$\begin{array}{ccc} & & \frac{d}{d\overset{ˇ}{x}}{𝛷}_{v,𝔫}(\overset{ˇ}{x})=-[{\hat{\lambda }}_{v}{\overset{´}{b}}_{v}+Q+\beta_{v}(\overset{ˇ}{x})]{𝛷}_{v,n}(\overset{ˇ}{x})+\hat{\lambda }\overset{´}{b}\sum_{k=1}^{n}X_{k}{𝛷}_{v,𝔫-k}(\overset{ˇ}{x})\\ & & \;\;\;+\int_{0}^{\infty }F_{w,𝔫}(\overset{ˇ}{x},z)\varphi_{w}(\overset{ˇ}{x})d\overset{ˇ}{x},𝔫\geq 1 \end{array}$$ (12)

$$\begin{array}{ccc} & & \frac{d}{dz}{\overset{ˇ}{\overset{ˇ}{R}}}_{ib,0}(\overset{ˇ}{x},z)=-[\hat{\lambda }\overset{´}{b}+\eta_{i}(z)]{\overset{ˇ}{\overset{ˇ}{R}}}_{ib,0}(\overset{ˇ}{x},z)+\hat{\lambda }(1-\overset{´}{b}){\overset{ˇ}{\overset{ˇ}{R}}}_{ib,0},𝔫=0,(i=1,2) \end{array}$$ (13)

$$\begin{array}{ccc} & & \frac{d}{dz}{\overset{ˇ}{\overset{ˇ}{R}}}_{ib,𝔫}(\overset{ˇ}{x},z)=-[\hat{\lambda }\overset{´}{b}+\eta_{i}(z)]{\overset{ˇ}{\overset{ˇ}{R}}}_{ib,𝔫}(\overset{ˇ}{x},z)+\hat{\lambda }\overset{´}{b}{\overset{ˇ}{\overset{ˇ}{R}}}_{ib,𝔫-1}(\overset{ˇ}{x},z)+\hat{\lambda }\overset{―}{b}{\overset{ˇ}{\overset{ˇ}{R}}}_{ib,𝔫}(\overset{ˇ}{x},z),𝔫\geq 1 \end{array}$$ (14)

$$\begin{array}{ccc} & & \frac{d}{dz}{\overset{ˇ}{\overset{ˇ}{R}}}_{r,0}(\overset{ˇ}{x},z)=-[\hat{\lambda }\overset{´}{b}+\eta_{r}(z)]{\overset{ˇ}{\overset{ˇ}{R}}}_{ir,0}(\overset{ˇ}{x},z)+\hat{\lambda }(1-\overset{´}{b}){\overset{ˇ}{\overset{ˇ}{R}}}_{ir,0}(\overset{ˇ}{x},z),𝔫=0 \end{array}$$ (15)

$$\begin{array}{ccc} & & \frac{d}{dz}{\overset{ˇ}{\overset{ˇ}{R}}}_{r,𝔫}(\overset{ˇ}{x},z)=-[\hat{\lambda }\overset{´}{b}+\eta_{r}(z)]{\overset{ˇ}{\overset{ˇ}{R}}}_{ir,𝔫}(\overset{ˇ}{x},z)+\hat{\lambda }\overset{´}{b}{\overset{ˇ}{\overset{ˇ}{R}}}_{ir,𝔫-1}(\overset{ˇ}{x},z)+\hat{\lambda }\overset{―}{b}{\overset{ˇ}{\overset{ˇ}{R}}}_{ir,𝔫}(\overset{ˇ}{x},z),𝔫\geq 1 \end{array}$$ (16)

$$\begin{array}{ccc} & & \frac{d}{dz}F_{w,0}(\overset{ˇ}{x},z)=-(\hat{\lambda }\overset{´}{b}+\varphi (z))F_{w,0}(\overset{ˇ}{x},z)+\hat{\lambda }(1-\overset{´}{b})F_{w,0}(\overset{ˇ}{x},z),𝔫=0 \end{array}$$ (17)

$$\begin{array}{ccc} & & \frac{d}{dz}F_{w,𝔫}(\overset{ˇ}{x},z)=-[{\hat{\lambda }}_{v}{\overset{´}{b}}_{v}+\varphi (z)]F_{w,𝔫}(\overset{ˇ}{x},z)+{\hat{\lambda }}_{v}{\overset{´}{b}}_{v}F_{w,𝔫-1}(\overset{ˇ}{x},z)+{\hat{\lambda }}_{v}\overset{―}{b_{v}}F_{w,𝔫}(\overset{ˇ}{x},z),𝔫\geq 1 \end{array}$$ (18)

At $\overset{ˇ}{x}=0$, and *z* = 0 the boundary conditions for a steady state system are provided as follows:


$$\begin{array}{ccc} T_{𝔫}(0) & = & \left(\begin{array}{c} (1-p)\omega_{1}\int_{0}^{\infty }{\tilde{S}}_{1b,𝔫}(\overset{ˇ}{x})\beta_{1b}(\overset{ˇ}{x})d\overset{ˇ}{x}\\ +(1-p)\int_{0}^{\infty }{\tilde{S}}_{2b,𝔫}(\overset{ˇ}{x})\beta_{2b}(\overset{ˇ}{x})d\overset{ˇ}{x}+\int_{0}^{\infty }\Omega_{ir,𝔫}(\overset{ˇ}{x})\beta_{ir}(\overset{ˇ}{x})d\overset{ˇ}{x}\\ +\int_{0}^{\infty }{𝛷}_{v,𝔫}(\overset{ˇ}{x})\beta_{v}(\overset{ˇ}{x})d\overset{ˇ}{x} \end{array}\right)\\ & & +\int_{0}^{\infty }F_{w,𝔫}(\overset{ˇ}{x},z)\varphi (z)dz,𝔫\geq 1, \end{array}$$ (19)

$$\begin{array}{ccc} {\tilde{S}}_{1b,0}(0) & = & \left(\begin{array}{c} \int_{0}^{\infty }T_{1}(\overset{ˇ}{x})\dot{a}(\overset{ˇ}{x})d\overset{ˇ}{x}+Q\int_{0}^{\infty }{𝛷}_{v,0}(\overset{ˇ}{x})d\overset{ˇ}{x}+\hat{\lambda }U_{0} \end{array}\right) \end{array}$$ (20)

$$\begin{array}{ccc} {\tilde{S}}_{1b,𝔫}(0) & = & \left(\begin{array}{c} \int_{0}^{\infty }T_{𝔫+1}(\overset{ˇ}{x})\dot{a}(\overset{ˇ}{x})d\overset{ˇ}{x}+\hat{\lambda }\overset{´}{b}\sum_{k=1}^{n}X_{k}\int_{0}^{\infty }T_{n-k+1}(\overset{ˇ}{x})d\overset{ˇ}{x}\\ +Q\int_{0}^{\infty }{𝛷}_{v,𝔫}(\overset{ˇ}{x})d\overset{ˇ}{x}+\hat{\lambda }X_{n+1}U_{0} \end{array}\right) \end{array}$$ (21)

$$\begin{array}{ccc} {\tilde{S}}_{2b,𝔫}(0) & = & \omega_{2}\int_{0}^{\infty }{\tilde{S}}_{1b,𝔫}(\overset{ˇ}{x})\beta_{1b}(\overset{ˇ}{x})d\overset{ˇ}{x} \end{array}$$ (22)

$$\begin{array}{ccc} \Omega_{r,𝔫}(0) & = & p\left(\begin{array}{c} \omega_{1}\int_{0}^{\infty }{\tilde{S}}_{1b,n}(\overset{ˇ}{x})\beta_{1}(\overset{ˇ}{x})d\overset{ˇ}{x}+\int_{0}^{\infty }{\tilde{S}}_{2b,n}(\overset{ˇ}{x})\beta_{2}(\overset{ˇ}{x})d\overset{ˇ}{x} \end{array}\right) \end{array}$$ (23)

$$\begin{array}{ccc} {𝛷}_{v,𝔫}(0) & = & \left\{ \begin{array}{c} \hat{\lambda }U_{0},\;𝔫=0\\ 0,\;𝔫\geq 1 \end{array}\right. \end{array}$$ (24)

$$\begin{array}{ccc} {\overset{ˇ}{\overset{ˇ}{R}}}_{ib,𝔫}(\overset{ˇ}{x},0) & = & \delta \sum_{𝔦=1}^{2}\int_{0}^{\infty }{\tilde{S}}_{ib,𝔫}(\overset{ˇ}{x})d\overset{ˇ}{x},(i=1,2) \end{array}$$ (25)

$$\begin{array}{ccc} {\overset{ˇ}{\overset{ˇ}{R}}}_{ir,𝔫}(\overset{ˇ}{x},0) & = & \delta \int_{0}^{\infty }\Omega_{r,𝔫}(\overset{ˇ}{x})d\overset{ˇ}{x} \end{array}$$ (26)

$$\begin{array}{ccc} F_{w,𝔫}(\overset{ˇ}{x},0) & = & \delta_{v}\int_{0}^{\infty }{𝛷}_{v,n}(\overset{ˇ}{x})d\overset{ˇ}{x},𝔫\geq 0 \end{array}$$ (27)

Here, provide the normalizing conditions for the proposed model


$$\begin{array}{ccc} & & U_{0}+\sum_{𝔫=1}^{\infty }\int_{0}^{\infty }T_{𝔫}(\overset{ˇ}{x})d\overset{ˇ}{x}+\sum_{𝔫=0}^{\infty }\left(\begin{array}{c} \sum_{𝔦=1}^{2}\int_{0}^{\infty }{\tilde{S}}_{ib,𝔫}(\overset{ˇ}{x})d\overset{ˇ}{x}+\int_{0}^{\infty }{𝛷}_{v,𝔫}(\overset{ˇ}{x})d\overset{ˇ}{x}+\int_{0}^{\infty }F_{w,𝔫}(\overset{ˇ}{x},z)d\overset{ˇ}{x}dz\\ +\sum_{𝔦=1}^{2}\int_{0}^{\infty }\int_{0}^{\infty }{\overset{ˇ}{\overset{ˇ}{R}}}_{ib,𝔫}(\overset{ˇ}{x},z)d\overset{ˇ}{x}dz+\sum_{𝔦=1}^{2}\int_{0}^{\infty }\int_{0}^{\infty }{\overset{ˇ}{\overset{ˇ}{R}}}_{r,𝔫}(\overset{ˇ}{x},z)d\overset{ˇ}{x}dz+\int_{0}^{\infty }\Omega_{r,𝔫}(\overset{ˇ}{x})d\overset{ˇ}{x} \end{array}\right)=1 \end{array}$$ (28)

### 3.2 Steady-state solution

We use the supplementary variable approach to obtain the equations of the system presented below. The structure of this system determines its performance. By using the probability generating function (PGF) approach, we derive the steady-state equations of the proposed retrial queueing model. Accordingly, the PGFs used to solve the governing equations are defined for |σ|≤1 as follows.

The obtained expressions reveal that the system behavior is governed by the interaction between retrial intensity, service efficiency, and server reliability. In particular, the denominator of the generating function reflects the stability balance between the arrival rate and the effective service rate, while the numerator captures the combined effects of retrial and optional re-service mechanisms. This structure provides valuable insight into how congestion develops under conditions of high retrial intensity or frequent server breakdowns.


$$\begin{array}{ccc} & & T(\overset{ˇ}{x},\;\sigma )=\sum_{𝔫=1}^{\infty }T_{𝔫}(\overset{ˇ}{x})\;\sigma^{𝔫};\;T(0,\;\sigma )=\sum_{𝔫=1}^{\infty }T_{𝔫}(0)\;\sigma^{𝔫}\\ & & {\tilde{S}}_{ib}(\overset{ˇ}{x},\;\sigma )=\sum_{𝔫=1}^{\infty }{\tilde{S}}_{ib,𝔫}(\overset{ˇ}{x})\;\sigma^{𝔫};\;{\tilde{S}}_{ib}(0,\;\sigma )=\sum_{𝔫=1}^{\infty }{\tilde{S}}_{ib,𝔫}(0)\;\sigma^{𝔫}\\ & & \Omega_{r}(\overset{ˇ}{x},\;\sigma )=\sum_{𝔫=1}^{\infty }\Omega_{r,𝔫}(\overset{ˇ}{x})\;\sigma^{𝔫};\;\Omega_{r}(0,\;\sigma )=\sum_{𝔫=1}^{\infty }\Omega_{r,𝔫}(0)\;\sigma^{𝔫}\\ & & {𝛷}_{v}(\overset{ˇ}{x},\;\sigma )=\sum_{𝔫=1}^{\infty }{𝛷}_{v,𝔫}(\overset{ˇ}{x})\;\sigma^{𝔫};\;{𝛷}_{v}(0,\;\sigma )=\sum_{𝔫=1}^{\infty }{𝛷}_{v,𝔫}(0)\;\sigma^{𝔫}\\ & & {\overset{ˇ}{\overset{ˇ}{R}}}_{ib}(\overset{ˇ}{x},z,\sigma )=\sum_{𝔫=1}^{\infty }{\overset{ˇ}{\overset{ˇ}{R}}}_{ib,𝔫}(\overset{ˇ}{x},z)\sigma^{𝔫};{\overset{ˇ}{\overset{ˇ}{R}}}_{ib}(\overset{ˇ}{x},0,\sigma )=\sum_{𝔫=1}^{\infty }{\overset{ˇ}{\overset{ˇ}{R}}}_{ib,𝔫}(\overset{ˇ}{x},0)\sigma^{𝔫}\;\\ & & {\overset{ˇ}{\overset{ˇ}{R}}}_{ir}(\overset{ˇ}{x},z,\sigma )=\sum_{𝔫=1}^{\infty }{\overset{ˇ}{\overset{ˇ}{R}}}_{ir,𝔫}(\overset{ˇ}{x},z)\;\sigma^{𝔫};{\overset{ˇ}{\overset{ˇ}{R}}}_{ir}(\overset{ˇ}{x},0,\sigma )=\sum_{𝔫=1}^{\infty }{\overset{ˇ}{\overset{ˇ}{R}}}_{ir,𝔫}(\overset{ˇ}{x},0)\;\sigma^{𝔫}\;\\ & & F_{w}(\overset{ˇ}{x},z,\sigma )=\sum_{𝔫=1}^{\infty }F_{w,𝔫}(\overset{ˇ}{x},z)\sigma^{𝔫};F_{w}(\overset{ˇ}{x},0,\sigma )=\sum_{𝔫=1}^{\infty }F_{w,𝔫}(\overset{ˇ}{x},0)\sigma^{𝔫}\;\;\; \end{array}$$

Where, [*i* = 1, 2]

The previously given PDE was obtained by multiplying the Eqs (1)–(27) by $\sigma^{𝔫}$ and subsequently summing over 𝔫, we get


$$\begin{array}{cc} & \frac{\partial }{\partial \overset{ˇ}{x}}T(\overset{ˇ}{x},\sigma )=-[\hat{\lambda }+\dot{a}(\overset{ˇ}{x})]T(\overset{ˇ}{x},\sigma ) \end{array}$$ (29)

$$\begin{array}{cc} & \frac{\partial }{\partial \overset{ˇ}{x}}{\tilde{S}}_{ib}(\overset{ˇ}{x},\sigma )=-[\hat{\lambda }\overset{´}{b}(1-X(\sigma ))+\delta +\beta_{ib}(\overset{ˇ}{x})]{\tilde{S}}_{ib}(\overset{ˇ}{x},\sigma ) \end{array}$$ (30)

$$\begin{array}{cc} & \frac{\partial }{\partial \overset{ˇ}{x}}\Omega_{r}(\overset{ˇ}{x},\sigma )=-[\hat{\lambda }\overset{´}{b}(1-X(\sigma ))+\delta +\beta_{r}(\overset{ˇ}{x})]\Omega_{r}(\overset{ˇ}{x},\sigma ) \end{array}$$ (31)

$$\begin{array}{cc} & \frac{\partial }{\partial \overset{ˇ}{x}}{𝛷}_{v}(\overset{ˇ}{x},\sigma )=-[{\hat{\lambda }}_{v}(1-X(\sigma )){\overset{´}{b}}_{v}+Q+\delta_{v}+\beta_{v}(\overset{ˇ}{x})]{𝛷}_{v}(\overset{ˇ}{x},\sigma ) \end{array}$$ (32)

$$\begin{array}{cc} & \frac{\partial }{\partial z}{\overset{ˇ}{\overset{ˇ}{R}}}_{ib}(\overset{ˇ}{x},z,\sigma )=-[\hat{\lambda }\overset{´}{b}(1-X(\sigma ))+\eta_{ib}(z)]{\overset{ˇ}{\overset{ˇ}{R}}}_{ib}(\overset{ˇ}{x},z,\sigma ) \end{array}$$ (33)

$$\begin{array}{cc} & \frac{\partial }{\partial z}{\overset{ˇ}{\overset{ˇ}{R}}}_{r}(\overset{ˇ}{x},z,\sigma )=-[\hat{\lambda }\overset{´}{b}(1-X(\sigma ))+\eta_{r}(z)]{\overset{ˇ}{\overset{ˇ}{R}}}_{r}(\overset{ˇ}{x},z,\sigma ) \end{array}$$ (34)

$$\begin{array}{cc} & \frac{\partial }{\partial z}F_{w}(\overset{ˇ}{x},z,\sigma )=-[{\hat{\lambda }}_{v}{\overset{´}{b}}_{v}(1-X(\sigma ))+\varphi (z)]F_{w}(\overset{ˇ}{x},z,\sigma ) \end{array}$$ (35)

$$\begin{array}{ccc} & & T(0,\sigma )=\left(\begin{array}{c} (1-p)\omega_{1}\int_{0}^{\infty }{\tilde{S}}_{1b}(\overset{ˇ}{x},\sigma )\beta_{1b}(\overset{ˇ}{x})d\overset{ˇ}{x}+(1-p)\int_{0}^{\infty }{\tilde{S}}_{2b}(\overset{ˇ}{x},\sigma )\beta_{2b}(\overset{ˇ}{x})d\overset{ˇ}{x}\\ +\int_{0}^{\infty }\Omega_{r}(\overset{ˇ}{x},\sigma )\beta_{2b}(\overset{ˇ}{x})d\overset{ˇ}{x}+\int_{0}^{\infty }{𝛷}_{v}(\overset{ˇ}{x},\sigma )\beta_{v}(\overset{ˇ}{x})d\overset{ˇ}{x}-\hat{\lambda }\overset{´}{b}U_{0} \end{array}\right) \end{array}$$ (36)

$$\begin{array}{ccc} & & {\tilde{S}}_{1b}(0,\sigma )=\frac{1}{\sigma }\int_{0}^{\infty }T(\overset{ˇ}{x},\sigma )\dot{a}(\overset{ˇ}{x})d\overset{ˇ}{x}+\hat{\lambda }X(\sigma )/\sigma \int_{0}^{\infty }T(\overset{ˇ}{x},\sigma )d\overset{ˇ}{x}+Q\int_{0}^{\infty }{𝛷}_{v}(\overset{ˇ}{x},\sigma )d\overset{ˇ}{x} \end{array}$$ (37)

$$\begin{array}{ccc} & & {\tilde{S}}_{2b}(0,\sigma )=\omega_{2}\int_{0}^{\infty }{\tilde{S}}_{1b}(\overset{ˇ}{x},\sigma )d\overset{ˇ}{x} \end{array}$$ (38)

$$\begin{array}{ccc} & & \Omega_{r,𝔫}(0,\sigma )=p\left(\begin{array}{c} \omega_{1}\int_{0}^{\infty }{\tilde{S}}_{1b,n}(\overset{ˇ}{x},\sigma )\beta_{1}(\overset{ˇ}{x})d\overset{ˇ}{x}+\int_{0}^{\infty }{\tilde{S}}_{2b,n}(\overset{ˇ}{x},\sigma )\beta_{2}(\overset{ˇ}{x})d\overset{ˇ}{x} \end{array}\right) \end{array}$$ (39)

$$\begin{array}{ccc} & & {𝛷}_{v}(0,\sigma )={\hat{\lambda }}_{v}U_{0} \end{array}$$ (40)

$$\begin{array}{ccc} & & {\overset{ˇ}{\overset{ˇ}{R}}}_{1b}(\overset{ˇ}{x},0,\sigma )=\delta \int_{0}^{\infty }{\tilde{S}}_{1b}(\overset{ˇ}{x},\sigma )d\overset{ˇ}{x} \end{array}$$ (41)

$$\begin{array}{ccc} & & {\overset{ˇ}{\overset{ˇ}{R}}}_{2b}(\overset{ˇ}{x},0,\sigma )=\delta \int_{0}^{\infty }{\tilde{S}}_{2b}(\overset{ˇ}{x},\sigma )d\overset{ˇ}{x} \end{array}$$ (42)

$$\begin{array}{ccc} & & {\overset{ˇ}{\overset{ˇ}{R}}}_{r}(\overset{ˇ}{x},0,\sigma )=\delta \int_{0}^{\infty }\Omega_{r}(\overset{ˇ}{x},\sigma )d\overset{ˇ}{x} \end{array}$$ (43)

$$\begin{array}{ccc} & & F_{w,𝔫}(\overset{ˇ}{x},0,\sigma )=\delta_{v}\int_{0}^{\infty }{𝛷}_{v}(\overset{ˇ}{x},\sigma )d\overset{ˇ}{x} \end{array}$$ (44)

By addressing the partial differential equations from Eq. (29) to Eq. (35), we get the following results.


$$\begin{array}{cc} & T(\overset{ˇ}{x},\sigma )=T(0,\sigma )[1-\hat{A}(\overset{ˇ}{x})]exp\left\{-\hat{\lambda }\overset{ˇ}{x}\right\} \end{array}$$ (45)

$$\begin{array}{cc} & {\tilde{S}}_{ib}(\overset{ˇ}{x},\sigma )={\tilde{S}}_{ib}(0,\sigma )\left[1-{\tilde{\tilde{𝕄}}}_{1b}(\overset{ˇ}{x})\right]exp\left\{-A_{ib}(\sigma )\overset{ˇ}{x}\right\} \end{array}$$ (46)

$$\begin{array}{cc} & \Omega_{r}(\overset{ˇ}{x},\sigma )=\Omega_{r}(0,\sigma )\left[1-{\tilde{\tilde{𝕄}}}_{r}(\overset{ˇ}{x})\right]exp\left\{-A_{r}(\sigma )\overset{ˇ}{x}\right\} \end{array}$$ (47)

$$\begin{array}{cc} & {𝛷}_{v}(\overset{ˇ}{x},\sigma )={𝛷}_{v}(0,\sigma )\left[1-{\tilde{\tilde{M}}}_{v}(\overset{ˇ}{x})\right]exp\left\{-A_{v}(\sigma )\overset{ˇ}{x}\right\} \end{array}$$ (48)

$$\begin{array}{cc} & F_{w}(\overset{ˇ}{x},z,\sigma )=F_{w}(\overset{ˇ}{x},0,\sigma )\left[1-G_{w}(\overset{ˇ}{x})\right]exp\left\{-\tau (\sigma )z\right\} \end{array}$$ (49)

$$\begin{array}{cc} & {\overset{ˇ}{\overset{ˇ}{R}}}_{1b}(\overset{ˇ}{x},z,\sigma )={\overset{ˇ}{\overset{ˇ}{R}}}_{1b}(\overset{ˇ}{x},0,\sigma )\left[1-W_{1}(z)\right]exp\left\{-\tau (\sigma )z\right\} \end{array}$$ (50)

$$\begin{array}{cc} & {\overset{ˇ}{\overset{ˇ}{R}}}_{2b}(\overset{ˇ}{x},z,\sigma )={\overset{ˇ}{\overset{ˇ}{R}}}_{2b}(\overset{ˇ}{x},0,\sigma )\left[1-W_{2}(z)\right]exp\left\{-\tau (\sigma )z\right\} \end{array}$$ (51)

$$\begin{array}{cc} & {\overset{ˇ}{\overset{ˇ}{R}}}_{r}(\overset{ˇ}{x},z,\sigma )={\overset{ˇ}{\overset{ˇ}{R}}}_{r}(\overset{ˇ}{x},0,\sigma )\left[1-W_{r}(z)\right]exp\left\{-\tau (\sigma )z\right\} \end{array}$$ (52)

By incorporating Eqs (45) and (48) in Eq (37) and subsequently making a few modifications, we get the following result,


$$\begin{array}{c} {\tilde{S}}_{1b}(0,\;\sigma )=\left(T(0,\;\sigma )/\sigma \right)\left(\begin{array}{c} {\hat{A}}^{*}(\hat{\lambda })+X(\sigma )\left(1-{\hat{A}}^{*}(\hat{\lambda })\right) \end{array}\right)+{\hat{\lambda }}_{v}U_{0}V(\sigma ) \end{array}$$ (53)

By substituting Eq (46) into Eq (38) and making a few modifications, we obtain the following result,


$$\begin{array}{c} {\tilde{S}}_{2b}(0,\;\sigma )=\omega_{2}{\tilde{S}}_{1b}(0,\;\sigma ){\tilde{\tilde{𝕄}}}_{1b}^{*}(A_{1b}(\sigma )) \end{array}$$ (54)

Substituting Eqs (46)–(48) into Eq (36), we obtain


$$\begin{array}{c} T(0,\;\sigma )=\left(\begin{array}{c} \sum_{i=1}^{2}{\tilde{S}}_{ib}(0,\;\sigma )\;{\tilde{\tilde{𝕄}}}_{1b}^{*}\left(A_{b}(\sigma )\right)+\Omega_{r}(0,\;\sigma )\;{\tilde{\tilde{𝕄}}}_{r}^{*}(A_{r}(\sigma ))+{𝛷}_{v}(0,\;\sigma )\;{\tilde{\tilde{M}}}_{v}^{*}\left(A_{v}(\sigma )\right) \end{array}\right) \end{array}$$ (55)

Substituting Eq (46) into Eq (39), we obtain


$$\begin{array}{c} \Omega_{r}(0,\;\sigma )=\left(\begin{array}{c} \sum_{i=1}^{2}{\tilde{S}}_{ib}(0,\;\sigma )\;{\tilde{\tilde{𝕄}}}_{ib}^{*}\left(A_{ib}(\sigma )\right) \end{array}\right) \end{array}$$ (56)

Using Eq (46) in Eq (41), we get


$$\begin{array}{c} {\overset{ˇ}{\overset{ˇ}{R}}}_{1b}(\overset{ˇ}{x},0,\;\sigma )={\tilde{S}}_{1b}(0,\;\sigma )\left(\frac{1-{\tilde{\tilde{𝕄}}}_{1b}^{*}\left(A_{b}(\sigma )\right)}{A_{1b}(\sigma )}\right) \end{array}$$ (57)

Using Eq (46) in Eq (42), we get


$$\begin{array}{c} {\overset{ˇ}{\overset{ˇ}{R}}}_{2b}(\overset{ˇ}{x},0,\;\sigma )={\tilde{S}}_{2b}(0,\;\sigma )\left(\frac{1-{\tilde{\tilde{𝕄}}}_{2b}^{*}\left(A_{b}(\sigma )\right)}{A_{2b}(\sigma )}\right) \end{array}$$ (58)

Using Eq (47) in Eq (43), we get


$$\begin{array}{c} {\overset{ˇ}{\overset{ˇ}{R}}}_{r}(\overset{ˇ}{x},0,\;\sigma )=\Omega_{r}(0,\;\sigma )\left(\frac{1-{\tilde{\tilde{𝕄}}}_{r}^{*}\left(A_{r}(\sigma )\right)}{A_{r}(\sigma )}\right) \end{array}$$ (59)

Using Eq (49) into Eq (44), we obtain


$$\begin{array}{c} F_{w}(\overset{ˇ}{x},0,\;\sigma )={𝛷}_{v}(0,\sigma )\delta_{v}{\hat{\lambda }}_{v}\left(\frac{1-{\tilde{\tilde{M}}}_{v}^{*}\left(A_{v}(\sigma )\right)}{A_{v}(\sigma )}\right) \end{array}$$ (60)

Using Eqs (53), (54) and (40) in Eq (55), we get


$$\begin{array}{c} T(0,\;\sigma )=\frac{\mathrm{Nu}(\sigma )}{\mathrm{De}(\sigma )} \end{array}$$ (61)

$$\begin{array}{c} \mathrm{Nu}(\sigma )=\sigma U_{0}\times \begin{array}{c} {\hat{\lambda }}_{v}\left(\begin{array}{c} \left((\omega_{1}+\omega_{2}\;{\tilde{\tilde{𝕄}}}_{2b}^{*}(A_{2b}(\sigma ))){\tilde{\tilde{𝕄}}}_{1b}(A_{1b}(\sigma ))V(\sigma )\right)\left(\overset{―}{p}+p{\tilde{\tilde{𝕄}}}_{r}^{*}\left(A_{r}(\sigma )\right)\right)\\ +{\tilde{\tilde{M}}}_{v}^{*}\left(A_{v}(\sigma )\right)-1 \end{array}\right) \end{array}\;\\ \mathrm{De}(\sigma )=\sigma -\left(\begin{array}{c} \left(\left[\begin{array}{c} {\hat{A}}^{*}(\hat{\lambda })+X(\sigma )(1-{\hat{A}}^{*}(\hat{\lambda })) \end{array}\right](\omega_{1}+\omega_{2}{\tilde{\tilde{𝕄}}}_{2b}^{*}(A_{2b}(\sigma ))){\tilde{\tilde{𝕄}}}_{1b}(A_{1b}(\sigma ))\right)\\ \left(\overset{―}{p}+p{\tilde{\tilde{𝕄}}}_{r}^{*}\left(A_{r}(\sigma )\right)\right) \end{array}\right)\; \end{array}$$

Using Eq (61) in Eq (53), we get


$$\begin{array}{c} {\tilde{S}}_{1b}(0,\;\sigma )=\frac{P_{0}}{De(\sigma )}\left\{\begin{array}{c} {\hat{\lambda }}_{v}\left(\begin{array}{c} {\tilde{\tilde{M}}}_{v}^{*}\left(A_{v}(\sigma )\right)-1 \end{array}\right)\left(\begin{array}{c} {\hat{A}}^{*}(\hat{\lambda })+X(\sigma )(1-{\hat{A}}^{*}(\hat{\lambda })) \end{array}\right)+\sigma V(\sigma ) \end{array}\right\} \end{array}$$ (62)

Using Eq (62) in Eq (54), we get


$$\begin{array}{c} {\tilde{S}}_{2b}(0,\;\sigma )=\frac{\omega_{2}{\tilde{\tilde{𝕄}}}_{1b}(A_{1b}(\sigma ))U_{0}}{De(\sigma )}\times \left\{\begin{array}{c} {\hat{\lambda }}_{v}\left(\begin{array}{c} {\tilde{\tilde{M}}}_{v}^{*}\left(A_{v}(\sigma )\right)-1 \end{array}\right)\\ \left({\hat{A}}^{*}(\hat{\lambda })+X(\sigma )(1-{\hat{A}}^{*}(\hat{\lambda })))+\sigma V(\sigma \right) \end{array}\right\} \end{array}$$ (63)

Using Eqs (62) and (63) in Eq (56), we get


$$\begin{array}{c} \Omega_{r}(0,\;\sigma )=\frac{p{\tilde{\tilde{𝕄}}}_{r}(A_{r}(\sigma ))U_{0}}{De(\sigma )}\times \left\{\begin{array}{c} {\hat{\lambda }}_{v}\left(\begin{array}{c} {\tilde{\tilde{M}}}_{v}^{*}\left(A_{v}(\sigma )\right)-1 \end{array}\right)\\ \left({\hat{A}}^{*}(\hat{\lambda })+X(\sigma )(1-{\hat{A}}^{*}(\hat{\lambda })))+\sigma V(\sigma \right) \end{array}\right\} \end{array}$$ (64)

Using the Eq (62) in Eq (57), we get


$$\begin{array}{c} {\overset{ˇ}{\overset{ˇ}{R}}}_{1b}(\overset{ˇ}{x},0,\;\sigma )=\frac{U_{0}\left(1-{\tilde{\tilde{𝕄}}}_{1b}^{*}\left(A_{1b}(\sigma )\right)\right)}{A_{1b}(\sigma )\times \mathrm{De}(\sigma )}\times \left\{\begin{array}{c} {\hat{\lambda }}_{v}\left(\begin{array}{c} {\tilde{\tilde{M}}}_{v}^{*}\left(A_{v}(\sigma )\right)-1 \end{array}\right)\\ \left({\hat{A}}^{*}(\hat{\lambda })+X(\sigma )(1-{\hat{A}}^{*}(\hat{\lambda })))+\sigma V(\sigma \right) \end{array}\right\} \end{array}$$ (65)

Using the Eq (63) in Eq (58), we get


$$\begin{array}{c} {\overset{ˇ}{\overset{ˇ}{R}}}_{2b}(\overset{ˇ}{x},0,\;\sigma )=\frac{U_{0}\left(1-{\tilde{\tilde{𝕄}}}_{2b}^{*}\left(A_{2b}(\sigma )\right)\right)}{A_{2b}(\sigma )\times \mathrm{De}(\sigma )}\times \left\{\begin{array}{c} {\hat{\lambda }}_{v}\left(\begin{array}{c} {\tilde{\tilde{M}}}_{v}^{*}\left(A_{v}(\sigma )\right)-1 \end{array}\right)\\ \left({\hat{A}}^{*}(\hat{\lambda })+X(\sigma )(1-{\hat{A}}^{*}(\hat{\lambda })))+\sigma V(\sigma \right) \end{array}\right\} \end{array}$$ (66)

Using the Eq (64) in Eq (59), we get


$$\begin{array}{c} {\overset{ˇ}{\overset{ˇ}{R}}}_{r}(\overset{ˇ}{x},0,\;\sigma )=\frac{U_{0}\left(1-{\tilde{\tilde{𝕄}}}_{r}^{*}\left(A_{r}(\sigma )\right)\right)}{A_{r}(\sigma )\times \mathrm{De}(\sigma )}\times \left\{\begin{array}{c} {\hat{\lambda }}_{v}\left(\begin{array}{c} {\tilde{\tilde{M}}}_{v}^{*}\left(A_{v}(\sigma )\right)-1 \end{array}\right)\\ \left({\hat{A}}^{*}(\hat{\lambda })+X(\sigma )(1-{\hat{A}}^{*}(\hat{\lambda })))+\sigma V(\sigma \right) \end{array}\right\} \end{array}$$ (67)

Using the Eq (40) in Eq (60), we get


$$\begin{array}{c} F_{w}(\overset{ˇ}{x},0,\;\sigma )=\delta_{v}{\hat{\lambda }}_{v}U_{0}\times \frac{1-{\tilde{\tilde{M}}}_{v}^{*}(A_{v}(\sigma ))}{A_{v}(\sigma )} \end{array}$$ (68)

**Theorem 2.** *The system is stabilizing under condition* ρ<1*; the average number of customers within a specific orbit and server state indicates whether the server is inactive, engaged in its usual FPS and SPS services, on vacation, or undergoing maintenance (FPS, SPS, re-service, and WV), respectively. Listed below is a compilation of generating functions:* $T(\sigma ),{\tilde{S}}_{1b}(\sigma ),{\tilde{S}}_{2b}(\sigma ),\Omega_{r}(\sigma ),{𝛷}_{v}(\sigma ),{\overset{ˇ}{\overset{ˇ}{R}}}_{1b}(\sigma ),{\overset{ˇ}{\overset{ˇ}{R}}}_{2b}(\sigma ),{\overset{ˇ}{\overset{ˇ}{R}}}_{r}(\sigma ),\text{and }F_{w}(\sigma )$*.*

*Proof.* We define the PGFs by integrating Eqs (45)–(52) with respect to $\overset{ˇ}{x}$,


$$\begin{array}{c} T(\sigma )=\int_{0}^{\infty }T(\overset{ˇ}{x},\sigma )d\overset{ˇ}{x}=\sigma U_{0}\left(\frac{1-{\hat{A}}^{*}(\hat{\lambda })}{\hat{\lambda }}\right)\times \frac{Nu2}{De1} \end{array}$$ (69)

$$\begin{array}{ccc} & & {\tilde{S}}_{1b}(\sigma )=\int_{0}^{\infty }{\tilde{S}}_{1b}(\overset{ˇ}{x},\sigma )d\overset{ˇ}{x}=\frac{{\hat{\lambda }}_{v}U_{0}(1-{\tilde{\tilde{𝕄}}}_{1b}^{*}(A_{1b}(\sigma )))}{A_{1b}(\sigma )}\times \frac{Nu1}{De1} \end{array}$$ (70)

$$\begin{array}{ccc} & & {\tilde{S}}_{2b}(\sigma )=\int_{0}^{\infty }{\tilde{S}}_{2b}(\overset{ˇ}{x},\sigma )d\overset{ˇ}{x}=\frac{\omega_{2}{\hat{\lambda }}_{v}U_{0}\left(\begin{array}{c} {\tilde{\tilde{𝕄}}}_{1b}^{*}(A_{1b}(\sigma ))\\ \left(1-{\tilde{\tilde{𝕄}}}_{2b}^{*}(A_{2b}(\sigma ))\right) \end{array}\right)}{A_{2b}(\sigma )}\times \frac{Nu1}{De1} \end{array}$$ (71)

$$\begin{array}{c} \Omega_{r}(\sigma )=\int_{0}^{\infty }\Omega_{r}(\overset{ˇ}{x},\sigma )d\overset{ˇ}{x}dz=\frac{p{\hat{\lambda }}_{v}U_{0}\left(\begin{array}{c} {1-\tilde{\tilde{𝕄}}}_{r}^{*}(A_{r}(\sigma )) \end{array}\right)}{A_{r}(\sigma )}\times \frac{Nu1}{De1} \end{array}$$ (72)

$$\begin{array}{c} {𝛷}_{v}(\sigma )=\int_{0}^{\infty }{𝛷}_{v}(\overset{ˇ}{x},\sigma )d\overset{ˇ}{x}={\hat{\lambda }}_{v}U_{0}V(\sigma )/Q\;\;\;\; \end{array}$$ (73)

$$\begin{array}{ccc} {\overset{ˇ}{\overset{ˇ}{R}}}_{1b}(\sigma )=\int_{0}^{\infty }\int_{0}^{\infty }{\overset{ˇ}{\overset{ˇ}{R}}}_{1b}(\overset{ˇ}{x},z,\sigma )d\overset{ˇ}{x}dz & = & \frac{\delta {\hat{\lambda }}_{v}U_{0}\left(\begin{array}{c} \left(1-W_{1b}^{*}(b(\sigma ))\right)\\ \left(1-{\tilde{\tilde{𝕄}}}_{1b}^{*}(A_{1b}(\sigma ))\right) \end{array}\right)}{A_{1b}(\sigma )\tau (\sigma )}\times \frac{Nu1}{De1}\; \end{array}$$ (74)

$$\begin{array}{ccc} {\overset{ˇ}{\overset{ˇ}{R}}}_{2b}(\sigma )=\int_{0}^{\infty }\int_{0}^{\infty }{\overset{ˇ}{\overset{ˇ}{R}}}_{2b}(\overset{ˇ}{x},z,\sigma )d\overset{ˇ}{x}dz & = & \frac{\delta {\hat{\lambda }}_{v}U_{0}\left(\begin{array}{c} \left(1-W_{2b}^{*}(b(\sigma ))\right)\\ \left(1-{\tilde{\tilde{𝕄}}}_{2b}^{*}(A_{2b}(\sigma ))\right) \end{array}\right)}{A_{2b}(\sigma )\tau (\sigma )}\times \frac{Nu1}{De1} \end{array}$$ (75)

$$\begin{array}{c} {\overset{ˇ}{\overset{ˇ}{R}}}_{r}(\sigma )=\int_{0}^{\infty }\int_{0}^{\infty }{\overset{ˇ}{\overset{ˇ}{R}}}_{r}(\overset{ˇ}{x},z,\sigma )d\overset{ˇ}{x}dz,{\overset{ˇ}{\overset{ˇ}{R}}}_{r}(\sigma )=\frac{\delta p{\hat{\lambda }}_{v}U_{0}\left(\begin{array}{c} \left(1-W_{r}^{*}(b(\sigma ))\right) \end{array}\right)}{A_{r}(\sigma )\tau (\sigma )}\times \frac{Nu1}{De1} \end{array}$$ (76)

$$\begin{array}{c} F_{w}(\sigma )=\int_{0}^{\infty }\int_{0}^{\infty }F_{w}(\overset{ˇ}{x},z,\sigma )d\overset{ˇ}{x}dz=\delta_{v}{\hat{\lambda }}_{v}U_{0}\frac{\left(1-G_{w}(b(\sigma ))\right)}{\tau (\sigma )}\frac{V(\sigma )}{Q}\;\; \end{array}$$ (77)

where


$$\begin{array}{ccc} & & Nu1=\left\{\left(\begin{array}{c} \;\;\left(\begin{array}{c} {\tilde{\tilde{M}}}_{v}^{*}\left(A_{v}(\sigma )\right)-1\;\; \end{array}\right)\left[\;\;\begin{array}{c} {\hat{A}}^{*}(\hat{\lambda })+X(\sigma )(1-{\hat{A}}^{*}(\hat{\lambda }))\;\; \end{array}\right]+\sigma V(\sigma ) \end{array}\;\;\right)\right\}\\ & & Nu2=\left({\hat{\lambda }}_{v}\left(\;\;\begin{array}{c} \;\;\begin{array}{c} \left((\omega_{1}+\omega_{2}\;{\tilde{\tilde{𝕄}}}_{2b}^{*}(A_{2b}(\sigma ))){\tilde{\tilde{𝕄}}}_{1b}^{*}(A_{1b}(\sigma ))V(\sigma )\right)\\ \left(\overset{―}{p}+p{\tilde{\tilde{𝕄}}}_{r}^{*}(A_{r}(\sigma ))\right)+{\tilde{\tilde{M}}}_{v}^{*}\left(A_{v}(\sigma )\right)-1 \end{array} \end{array}\right)\right)\\ & & De1=\left\{\;\;\;\begin{array}{c} \sigma -\left[\begin{array}{c} {\hat{A}}^{*}(\hat{\lambda })+X(\sigma )(1-{\hat{A}}^{*}(\hat{\lambda })) \end{array}\right]\left(\begin{array}{c} \omega_{1}+\omega_{2}\;{\tilde{\tilde{𝕄}}}_{2b}^{*}(A_{2b}(\sigma )))\\ {\tilde{\tilde{𝕄}}}_{1b}^{*}(A_{1b}(\sigma ) \end{array}\right)\\ \left(\overset{―}{p}+p{\tilde{\tilde{𝕄}}}_{r}^{*}(A_{r}(\sigma ))\right) \end{array}\;\;\right\} \end{array}$$

$$\begin{array}{c} U_{0}=\frac{1-X1(1-{\hat{A}}^{*}(\hat{\lambda }))-P1}{\left\{\begin{array}{c} 1-X1(1-{\hat{A}}^{*}(\hat{\lambda })-P1)\\ +{\hat{\lambda }}_{v}{\overset{´}{b}}_{v}\left(E({\tilde{\tilde{𝕄}}}_{1b})+\omega_{2}E({\tilde{\tilde{𝕄}}}_{2b})\right)\left(\begin{array}{c} \left({\tilde{\tilde{M}}}_{v}^{*}(Q)-1)X1(1-{\hat{A}}^{*}(\hat{\lambda }))+(1-{\tilde{\tilde{M}}}_{v}^{*}(Q)\right)\\ -(1-{\tilde{\tilde{M}}}_{v}^{*}(Q))X1(-{\hat{\lambda }}_{v}{\overset{´}{b}}_{v}-{\hat{\lambda }}_{v}{\overset{´}{b}}_{v}\delta_{v}(g_{v}^{1})/Q) \end{array}\right)\times \\ \left(1+\delta (g^{1}))+\left(\begin{array}{c} {\hat{\lambda }}_{v}\left(\begin{array}{c} 1-X1(1-{\hat{A}}^{*}(\hat{\lambda }))-P1 \end{array}\right)(1-{\tilde{\tilde{M}}}_{v}^{*}(Q)/Q) \end{array}\right)\times (1+\delta_{v}(g_{v}^{1})\right)\\ +\left(\begin{array}{c} \;\;\;((1-{\tilde{\tilde{M}}}_{v}^{*}(Q))P1)+(1-{\tilde{\tilde{M}}}_{v}^{*}(Q))+(1-{\tilde{\tilde{M}}}_{v}^{*}(Q))X1({\hat{\lambda }}_{v}{\overset{´}{b}}_{v}+\delta ({\hat{\lambda }}_{v}{\overset{´}{b}}_{v}(g^{1}))/Q)\;\;\; \end{array}\right)\times (1-{\hat{A}}^{*}(\hat{\lambda })) \end{array}\right\}} \end{array}$$ (78)

We can calculate the probability that the server is idle (*U*_0_) by applying the normalized condition. By substituting σ=1 in Eqs (69)–(77) and utilizing the rule of L’Hospital’s whenever appropriate, we obtain


$$\begin{array}{c} \left(\;\;\begin{array}{c} U_{0}+T(1)+{\tilde{S}}_{1b}(1)+{\tilde{S}}_{2b}(1)+\Omega_{r}(1)+{𝛷}_{v}(1)+F_{w}(1)+{\overset{ˇ}{\overset{ˇ}{R}}}_{1b}(1)+{\overset{ˇ}{\overset{ˇ}{R}}}_{2b}(1)+{\overset{ˇ}{\overset{ˇ}{R}}}_{r}(1)\;\;\; \end{array}\right)=1 \end{array}$$

**Remark.** In the absence of re-service (i.e., *p* = 0), the proposed model reduces to a classical two-phase retrial queue with working vacation. This demonstrates that the present model generalizes existing results as a special case. □

**Theorem 3.** *The model must satisfy the stability criterion given by the equation* ρ<1*. The PGF of the number of customers appearing in both the system and an orbit was denoted by the functions* $K_{o}(\sigma )$ *and* $K_{s}(\sigma )$ *across various server states, such as idle, FPS, SPS, re-service, reduced rate service, and under maintenance.*


$$\begin{array}{ccc} K_{0}(\sigma ) & = & \left(\begin{array}{c} U_{0}+T(\sigma )+{\tilde{S}}_{1b}(\sigma )+{\tilde{S}}_{2b}(\sigma )+\Omega_{r}(\sigma )+{𝛷}_{v}(\sigma )+F_{w}(\sigma )\\ +{\overset{ˇ}{\overset{ˇ}{R}}}_{1b}(\sigma )+{\overset{ˇ}{\overset{ˇ}{R}}}_{2b}(\sigma )+{\overset{ˇ}{\overset{ˇ}{R}}}_{r}(\sigma ) \end{array}\right) \end{array}$$ (79)

as well as,


$$\begin{array}{ccc} K_{s}(\sigma ) & = & \left(\begin{array}{c} U_{0}+T(\sigma )+\sigma \left({\tilde{S}}_{1b}(\sigma )+{\tilde{S}}_{2b}(\sigma )+\Omega_{r}(\sigma )+{𝛷}_{v}(\sigma )\right)+F_{w}(\sigma )\\ +{\overset{ˇ}{\overset{ˇ}{R}}}_{1b}(\sigma )+{\overset{ˇ}{\overset{ˇ}{R}}}_{2b}(\sigma )+{\overset{ˇ}{\overset{ˇ}{R}}}_{r}(\sigma ) \end{array}\right) \end{array}$$ (80)

*Proof.* Using equations indicated as (69)-(78) as substitutes for Eq (79) $K_{0}(\sigma )=U_{0}+T(\sigma )+{\tilde{S}}_{1b}(\sigma )+{\tilde{S}}_{2b}(\sigma )+\Omega_{r}+{𝛷}_{v}(\sigma )\;+$${\overset{ˇ}{\overset{ˇ}{R}}}_{r}(\sigma )+F_{w}(\sigma )+{\overset{ˇ}{\overset{ˇ}{R}}}_{1b}(\sigma )+{\overset{ˇ}{\overset{ˇ}{R}}}_{2b}(\sigma )$. We have determined the PGF for the orbit’s length.,


$$\begin{array}{c} K_{0}(\sigma )=U_{0}\frac{Nu_{o}(\sigma )}{De_{s}(\sigma )} \end{array}$$ (81)

Where,


$$\begin{array}{c} Nu_{o}(\sigma )=Y_{1}+Y_{2}+Y_{3}+Y_{4} \end{array}$$

Furthermore, by modifying the equations via (69)-(78) into Eq (80) $K_{s}(\sigma )=U_{0}+T(\sigma )+\sigma \left({\tilde{S}}_{1b}(\sigma )+{\tilde{S}}_{2b}(\sigma )+{𝛷}_{v}(\sigma )\right)\;+$$F_{w}(\sigma )+{\overset{ˇ}{\overset{ˇ}{R}}}_{1b}(\sigma )+{\overset{ˇ}{\overset{ˇ}{R}}}_{2b}(\sigma )$, We have determined the PGF for the system’s length.


$$\begin{array}{c} K_{s}(\sigma )=U_{0}\frac{Nu_{s}(\sigma )}{De_{s}(\sigma )} \end{array}$$ (82)

Where, *U*_0_ mentioned by the Eq (78)


$$\begin{array}{c} Nu_{s}=Y_{1}+Y_{2}+Y_{5}+Y_{6} \end{array}$$

$\begin{array}{cc} Y_{1} & =\hat{\lambda }\overset{´}{b}(1-X(\sigma ))U_{0}\left\{\begin{array}{c} \sigma -\left[\begin{array}{c} {\hat{A}}^{*}(\hat{\lambda })+X(\sigma )(1-{\hat{A}}^{*}(\hat{\lambda })) \end{array}\right]\times Qu1 \end{array}\right\}\\ Y_{2} & =(1-{\hat{A}}^{*}(\hat{\lambda }))\sigma \hat{\lambda }\overset{´}{b}(1-X(\sigma ))U_{0}\left({\hat{\lambda }}_{v}\left(\begin{array}{c} \begin{array}{c} Qu1+{\tilde{\tilde{M}}}_{v}^{*}\left(A_{v}(\sigma )\right)-1 \end{array} \end{array}\right)\right)\\ Y_{3} & ={\hat{\lambda }}_{v}U_{0}Qu2\times \left(\begin{array}{c} \left(\begin{array}{c} {\tilde{\tilde{M}}}_{v}^{*}\left(A_{v}(\sigma )\right)-1 \end{array}\right)\left(\begin{array}{c} {\hat{A}}^{*}(\hat{\lambda })+X(\sigma )(1-{\hat{A}}^{*}(\hat{\lambda })) \end{array}\right)+\sigma V(\sigma ) \end{array}\right)\\ Y_{4} & =\frac{U_{0}{\hat{\lambda }}_{v}V(\sigma )}{Q}\times \left(\begin{array}{c} \hat{\lambda }\overset{´}{b}(1-X(\sigma ))\\ +\delta_{v}(1-G_{w}(\tau_{v}(\sigma ))) \end{array}\right)\left\{\begin{array}{c} \sigma -\left[\begin{array}{c} {\hat{A}}^{*}(\hat{\lambda })+X(\sigma )\\ \left(1-{\hat{A}}^{*}(\hat{\lambda })\right) \end{array}\right]\times Qu1 \end{array}\right\}\\ Y_{5} & =\sigma {\hat{\lambda }}_{v}U_{0}Qu2\times \left(\begin{array}{c} \left(\begin{array}{c} {\tilde{\tilde{M}}}_{v}^{*}\left(A_{v}(\sigma )\right)-1 \end{array}\right)\left(\begin{array}{c} {\hat{A}}^{*}(\hat{\lambda })+X(\sigma )(1-{\hat{A}}^{*}(\hat{\lambda })) \end{array}\right)+\sigma V(\sigma ) \end{array}\right)\\ Y_{6} & =\frac{U_{0}{\hat{\lambda }}_{v}V(\sigma )}{Q}\left(\begin{array}{c} \sigma \hat{\lambda }\overset{´}{b}(1-X(\sigma ))+\sigma \delta_{v}\\ \left(1-G_{w}(\tau_{v}(\sigma ))\right) \end{array}\right)\times \left\{\begin{array}{c} \sigma -\left[\begin{array}{c} {\hat{A}}^{*}(\hat{\lambda })+X(\sigma )\\ \left(1-{\hat{A}}^{*}(\hat{\lambda })\right) \end{array}\right]\times Qu1 \end{array}\right\}\\ De_{s}(\sigma ) & =\left\{\sigma -\left[\begin{array}{c} {\hat{A}}^{*}(\hat{\lambda })+X(\sigma )(1-{\hat{A}}^{*}(\hat{\lambda })) \end{array}\right]\times Qu1\right\}\\ Qu1 & =\left(\begin{array}{c} \omega_{1}+\omega_{2}\;{\tilde{\tilde{𝕄}}}_{2b}^{*}(A_{2b}(\sigma )){\tilde{\tilde{𝕄}}}_{1b}^{*}(A_{1b}(\sigma )) \end{array}\right)\left(\overset{―}{p}+p{\tilde{\tilde{𝕄}}}_{r}^{*}(A_{r}(\sigma ))\right)\\ Qu2 & =\left(\begin{array}{c} \left(1-{\tilde{\tilde{𝕄}}}_{1b}^{*}(A_{1b}(\sigma )))+(1-{\tilde{\tilde{𝕄}}}_{2b}^{*}(A_{2b}(\sigma )))\omega_{2}{\tilde{\tilde{𝕄}}}_{1b}^{*}(A_{1b}(\sigma )\right)\\ +(1-{\tilde{\tilde{𝕄}}}_{r}^{*}(A_{r}(\sigma ))) \end{array}\right) \end{array}$ □

## 4 System’s characteristic

This section formulates performance metrics for the proposed queuing system. The primary indicators developed include long-term probabilities, mean system and orbit sizes, server availability, and mean busy and busy cycle periods.

### 4.1 Probabilities of the system state

In a steady-state condition, the probabilities of various system states are obtained by setting σ→1 in the relevant marginal probability generating functions (PGFs) and applying L’Hopital’s rule. The following results are obtained from Eqs (69)–(77).

1. The possibility *T*(1) that the server will remain accessible throughout the duration of the repeated attempts is given by:
  $$\begin{array}{c} T(1)=U_{0}\left(1-{\hat{A}}^{*}(\hat{\lambda })\right)\times \left\{\frac{\begin{array}{c} \left(1-{\tilde{\tilde{M}}}_{v}^{*}(Q)\right)\\ +\left(\begin{array}{c} (1-{\tilde{\tilde{M}}}_{v}^{*}(Q))P1\\ +((1-{\tilde{\tilde{M}}}_{v}(Q)){\hat{\lambda }}_{v}{\overset{´}{b}}_{v}X1(1+\delta_{v}g_{v}^{1}))/Q \end{array}\right) \end{array}}{1-X1(1-{\hat{A}}^{*}(\hat{\lambda }))-P1}\right\} \end{array}$$ (83)
2. The probability ${\tilde{S}}_{1b}(1)$ that the server is occupied during the FPS is given below:
  $$\begin{array}{c} {\tilde{S}}_{1b}(1)={\hat{\lambda }}_{v}U_{0}\times \left\{\frac{E({\tilde{\tilde{𝕄}}}_{1b})\left(\begin{array}{c} (1-{\tilde{\tilde{M}}}_{v}^{*}(Q))+\left(\begin{array}{c} \left({\tilde{\tilde{M}}}_{v}^{*}(Q)-1\right)\\ X1(1-{\hat{𝔸}}^{*}(\hat{\lambda })) \end{array}\right)\\ +\left(\begin{array}{c} \left(1-{\tilde{\tilde{M}}}_{v}^{*}(Q)\right)\\ ({\hat{\lambda }}_{v}{\overset{´}{b}}_{v}X1(1+\delta_{v}g_{v}^{1}))/Q \end{array}\right) \end{array}\right)}{1-X1(1-{\hat{A}}^{*}(\hat{\lambda }))-P1}\right\} \end{array}$$ (84)
3. The probability ${\tilde{S}}_{2b}(1)$ that the server is occupied during the SPS of service is given by:
  $$\begin{array}{c} {\tilde{S}}_{2b}(1)=\omega_{2}{\hat{\lambda }}_{v}U_{0}\times \left\{\frac{E({\tilde{\tilde{𝕄}}}_{2b})\left(\begin{array}{c} (1-{\tilde{\tilde{M}}}_{v}^{*}(Q))+\left(\begin{array}{c} \left({\tilde{\tilde{M}}}_{v}^{*}(Q)-1\right)\\ X1(1-{\hat{𝔸}}^{*}(\hat{\lambda })) \end{array}\right)\\ +\left(\begin{array}{c} \left(1-{\tilde{\tilde{M}}}_{v}^{*}(Q)\right)\\ ({\hat{\lambda }}_{v}{\overset{´}{b}}_{v}X1(1+\delta_{v}g_{v}^{1}))/Q \end{array}\right) \end{array}\right)}{1-X1(1-{\hat{A}}^{*}(\hat{\lambda }))-P1}\right\} \end{array}$$ (85)
4. The probability ${\tilde{\tilde{𝕄}}}_{r}(1)$ that the server is occupied during the re-service is given by:
  $$\begin{array}{c} \Omega_{r}(1)=p{\hat{\lambda }}_{v}U_{0}\times \left\{\frac{E(\tilde{\tilde{{𝕄}_{r}}})\left(\begin{array}{c} (1-{\tilde{\tilde{M}}}_{v}^{*}(Q))+\left(\begin{array}{c} \left({\tilde{\tilde{M}}}_{v}^{*}(Q)-1\right)\\ X1(1-{\hat{A}}^{*}(\hat{\lambda })) \end{array}\right)\\ +\left(\begin{array}{c} \left(1-{\tilde{\tilde{M}}}_{v}^{*}(Q)\right)\\ ({\hat{\lambda }}_{v}{\overset{´}{b}}_{v}X1(1+\delta_{v}g_{v}^{1}))/Q \end{array}\right) \end{array}\right)}{1-X1(1-{\hat{A}}^{*}(\hat{\lambda }))-P1}\right\} \end{array}$$ (86)
5. The probability ${𝛷}_{v}(1)$ that the server functioning at reduced rate service is given below:
  $$\begin{array}{c} {𝛷}_{v}(1)=\left\{{\hat{\lambda }}_{v}U_{0}(1-{\tilde{\tilde{M}}}_{v}^{*}(Q))/Q\right\} \end{array}$$ (87)
6. The probability ${\overset{ˇ}{\overset{ˇ}{R}}}_{1b}(1)$ of the server undergoing maintenance during the FPS service is given below:
  $$\begin{array}{c} {\overset{ˇ}{\overset{ˇ}{R}}}_{1b}(1)=\delta {\hat{\lambda }}_{v}U_{0}g^{1f}\times \left\{\frac{E({\tilde{\tilde{𝕄}}}_{1b})\left(\begin{array}{c} (1-{\tilde{\tilde{M}}}_{v}^{*}(Q))+\left(\begin{array}{c} \left({\tilde{\tilde{M}}}_{v}^{*}(Q)-1\right)\\ X1(1-{\hat{A}}^{*}(\hat{\lambda })) \end{array}\right)\\ +\left(\begin{array}{c} \left(1-{\tilde{\tilde{M}}}_{v}^{*}(Q)\right)\\ ({\hat{\lambda }}_{v}{\overset{´}{b}}_{v}X1(1+\delta_{v}g_{v}^{1}))/Q \end{array}\right) \end{array}\right)}{1-X1(1-{\hat{A}}^{*}(\hat{\lambda }))-P1}\right\} \end{array}$$ (88)
7. The probability ${\overset{ˇ}{\overset{ˇ}{R}}}_{2b}(1)$ of the server undergoing maintenance during the SPS service is given by:
  $$\begin{array}{c} {\overset{ˇ}{\overset{ˇ}{R}}}_{2b}(1)=\omega_{2}\delta {\hat{\lambda }}_{v}U_{0}g^{1s}\times \left\{\frac{E({\tilde{\tilde{𝕄}}}_{2b})\left(\begin{array}{c} (1-{\tilde{\tilde{M}}}_{v}^{*}(Q))+\left(\begin{array}{c} \left({\tilde{\tilde{M}}}_{v}^{*}(Q)-1\right)\\ X1(1-{\hat{A}}^{*}(\hat{\lambda })) \end{array}\right)\\ +\left(\begin{array}{c} \left(1-{\tilde{\tilde{M}}}_{v}^{*}(Q)\right)\\ ({\hat{\lambda }}_{v}{\overset{´}{b}}_{v}X1(1+\delta_{v}g_{v}^{1}))/Q \end{array}\right) \end{array}\right)}{1-X1(1-{\hat{A}}^{*}(\hat{\lambda }))-P1}\right\} \end{array}$$ (89)
8. The probability ${\overset{ˇ}{\overset{ˇ}{R}}}_{r}(1)$ of the server undergoing maintenance during the re-service is given by:
  $$\begin{array}{c} {\overset{ˇ}{\overset{ˇ}{R}}}_{r}(1)=\delta {\hat{\lambda }}_{v}U_{0}g^{1r}\times \left\{\frac{E(\tilde{\tilde{{𝕄}_{r}}})\left(\begin{array}{c} (1-{\tilde{\tilde{M}}}_{v}^{*}(Q))+\left(\begin{array}{c} \left({\tilde{\tilde{M}}}_{v}^{*}(Q)-1\right)\\ X1(1-{\hat{A}}^{*}(\hat{\lambda })) \end{array}\right)\\ +\left(\begin{array}{c} \left(1-{\tilde{\tilde{M}}}_{v}^{*}(Q)\right)\\ ({\hat{\lambda }}_{v}{\overset{´}{b}}_{v}X1(1+\delta_{v}g_{v}^{1}))/Q \end{array}\right) \end{array}\right)}{1-X1(1-{\hat{A}}^{*}(\hat{\lambda }))-P1}\right\} \end{array}$$ (90)
9. The probability $F_{w}(1)$ of the server undergoing maintenance during WV period is given by:
  $$\begin{array}{c} F_{w}(1)=\delta_{v}{\hat{\lambda }}_{v}U_{0}g_{v}^{1}\frac{\left(1-{\tilde{\tilde{M}}}_{v}^{*}(Q)\right)}{Q} \end{array}$$ (91)

### 4.2 The average size of orbit and average size of system

We differentiate Eq (81) with respect to σ and calculate the number of customers in the orbit $\pi_{q}$ at σ=1; it is provided by


$$\begin{array}{c} \pi_{q}=K_{0}^{\prime }(1)=\lim_{\sigma \to 1}K_{0}^{\prime }(\sigma )=U_{0}\left[\frac{Nu_{o}^{\prime \prime \prime }(1)De_{s}^{\prime \prime }(1)-De_{s}^{\prime \prime \prime }(1)Nu_{o}^{\prime \prime }(1)}{3(De_{s}^{\prime \prime }(1){)}^{2}}\right] \end{array}$$ (92)

Also,

Differentiating Eq (82) with respect to σ=1 and computing the number of customers in the system $L_{s}$ at σ=1 is determined as follows.


$$\begin{array}{c} L_{s}=K_{s}^{\prime }(1)=\lim_{\sigma \to 1}K_{s}^{\prime }(\sigma )=U_{0}\left[\frac{Nu_{s}^{\prime \prime \prime }(1)De_{s}^{\prime \prime }(1)-De_{s}^{\prime \prime \prime }(1)Nu_{o}^{\prime \prime }(1)}{3(De_{s}^{\prime \prime }(1){)}^{2}}\right] \end{array}$$ (93)

It is possible to find the outcomes of the $\pi_{q}$ and $L_{s}$ calculations in S1 Appendix.

### 4.3 Analysis of the system’s reliability metric

To improve the predictability of a system vulnerable to failure, it is important to consider the reliability metrics of the model. These metrics provide significant insight into the mean availability of servers and other relevant indicators. We analyze and evaluate the framework of analytical results; the availability metric $S_{Av}$ and the failure occurrence metric $Fail_{f}$ are formulated as follows:

1. The server mean availability ($S_{Av}$) is provided by:
  $$\begin{array}{cc} S_{Av} & =1-\lim_{\sigma \to 1}({\overset{ˇ}{\overset{ˇ}{R}}}_{1b}(\sigma )+{\overset{ˇ}{\overset{ˇ}{R}}}_{2b}(\sigma )+{\overset{ˇ}{\overset{ˇ}{R}}}_{r}(\sigma )+F_{w}(\sigma ))\\ & =1-({\overset{ˇ}{\overset{ˇ}{R}}}_{1b}(1)+{\overset{ˇ}{\overset{ˇ}{R}}}_{2b}(1)+{\overset{ˇ}{\overset{ˇ}{R}}}_{r}(1)+F_{w}(1))\\ & =1-\left\{\begin{array}{c} \delta {\hat{\lambda }}_{v}U_{0}\left(g^{1}E(\tilde{\tilde{{𝕄}_{1b}}})+\omega_{2}g^{1}E({\tilde{\tilde{𝕄}}}_{1b})+g^{1}E(\tilde{\tilde{{𝕄}_{r}}})\right)\times \\ \left(\frac{\left(\begin{array}{c} \left(1-{\tilde{\tilde{M}}}_{v}^{*}(Q))+({\tilde{\tilde{M}}}_{v}^{*}(Q)-1)X1(1-{\hat{𝔸}}^{*}(\hat{\lambda })\right)\\ +\left(\begin{array}{c} (1-{\tilde{\tilde{M}}}_{v}^{*}(Q))({\hat{\lambda }}_{v}{\overset{´}{b}}_{v}X1(1+\delta_{v}g_{v}^{1}))/Q \end{array}\right) \end{array}\right)}{1-X1(1-{\hat{𝔸}}^{*}(\hat{\lambda }))-P1}\right)\\ +\delta_{v}{\hat{\lambda }}_{v}U_{0}g_{v}^{1}\frac{\left(1-{\tilde{\tilde{M}}}_{v}^{*}(Q)\right)}{Q} \end{array}\right\} \end{array}$$
2. The steady-state failure occurs in the following manner:
  $$\begin{array}{cc} Fail_{f} & =\delta \times \left({\tilde{S}}_{1b}(1)+{\tilde{S}}_{2b}(1)+\Omega_{r}(1)\right)+\delta_{v}\times {𝛷}_{v}(1)\\ & =\left\{\begin{array}{c} \delta {\hat{\lambda }}_{v}U_{0}\left(E({\tilde{\tilde{𝕄}}}_{1b})+\omega_{2}E({\tilde{\tilde{𝕄}}}_{2b})+E({\tilde{\tilde{𝕄}}}_{r})\right)\times \\ \left(\frac{\left(\begin{array}{c} \left(1-{\tilde{\tilde{M}}}_{v}^{*}(Q))+({\tilde{\tilde{M}}}_{v}^{*}(Q)-1)X1(1-{\hat{A}}^{*}(\hat{\lambda })\right)\\ +\left(\begin{array}{c} (1-{\tilde{\tilde{M}}}_{v}^{*}(Q))({\hat{\lambda }}_{v}{\overset{´}{b}}_{v}X1(1+\delta_{v}g_{v}^{1}))/Q \end{array}\right) \end{array}\right)}{1-X1(1-{\hat{A}}^{*}(\hat{\lambda }))-P1}\right)\\ +\delta_{v}{\hat{\lambda }}_{v}U_{0}\frac{\left(1-{\tilde{\tilde{M}}}_{v}^{*}(Q)\right)}{Q} \end{array}\right\} \end{array}$$

### 4.4 Length of the mean busy time and the mean busy cycle

We consider the mean busy cycle $ℍ({𝕋}_{bc})$, and the period of server busy is $ℍ({𝕋}_{bp})$. The results of this study derive directly from the concepts of an alternative renewal process and lead to the following conclusions.


$$\begin{array}{c} U_{0}=\frac{ℍ({𝕋}_{0})}{ℍ({𝕋}_{0})+ℍ({𝕋}_{bs})};ℍ({𝕋}_{bs})=\frac{1}{\hat{\lambda }}(\frac{1}{U_{0}}-1)\text{and}\\ ℍ({𝕋}_{bc})=\frac{1}{(\hat{\lambda })U_{0}}=ℍ({𝕋}_{0})+ℍ({𝕋}_{bs}) \end{array}$$ (94)

Where, ${𝕋}_{0}$ represents the system length in the unoccupied state, and the operator $H({𝕋}_{0})$ is defined as $\left(1/\hat{\lambda }\right)$. By substituting Eq (78) into Eq (94), we obtain the expected result as


$$\begin{array}{c} H({𝕋}_{bs})=\frac{1}{\hat{\lambda }}\frac{\left\{\begin{array}{c} {\hat{\lambda }}_{v}\left(E({\tilde{\tilde{𝕄}}}_{1b})+\omega_{2}E({\tilde{\tilde{𝕄}}}_{2b})+E({\tilde{\tilde{𝕄}}}_{r})\right)\times \\ \left(\begin{array}{c} \left(1-{\tilde{\tilde{M}}}_{v}^{*}(Q))+({\tilde{\tilde{M}}}_{v}^{*}(Q)-1)X1(1-{\hat{A}}^{*}(\hat{\lambda })\right)\\ -(1-{\tilde{\tilde{M}}}_{v}^{*}(Q))X1(-{\hat{\lambda }}_{v}{\overset{´}{b}}_{v}-{\hat{\lambda }}_{v}{\overset{´}{b}}_{v}\delta_{v}(g_{v}^{1})/Q) \end{array}\right)\times (1+\delta (g^{1}))\\ +\left(\begin{array}{c} {\hat{\lambda }}_{v}\left(\begin{array}{c} 1-X1(1-{\hat{A}}^{*}(\hat{\lambda }))-P1 \end{array}\right)(1-{\tilde{\tilde{𝕄}}}_{1b}^{*}(Q)/Q) \end{array}\right)\times (1+\delta_{v}(g_{v}^{1}))\\ +\left((1-{\tilde{\tilde{M}}}_{v}^{*}(Q))+\left(\begin{array}{c} (1-{\tilde{\tilde{M}}}_{v}^{*}(Q))P1\\ +({\hat{\lambda }}_{v}{\overset{´}{b}}_{v}X1(1-{\tilde{\tilde{M}}}_{v}^{*}(Q))(1+\delta_{v}g_{v}^{1}))/Q \end{array}\right)\right)\times (1-{\hat{A}}^{*}(\hat{\lambda })) \end{array}\right\}}{1-X1(1-{\hat{A}}^{*}(\hat{\lambda })-P1)} \end{array}$$

$$\begin{array}{c} H\left({𝕋}_{bc}\right)=\frac{{\hat{\lambda }}^{-1}\left\{\begin{array}{c} 1-X1(1-{\hat{A}}^{*}(\hat{\lambda })-P1)\\ +\left(\begin{array}{c} \left(1-{\tilde{\tilde{M}}}_{v}^{*}(Q)\right)\\ +\left((1-{\tilde{\tilde{M}}}_{v}^{*}(Q))P1+({\hat{\lambda }}_{v}{\overset{´}{b}}_{v}X1(1-{\tilde{\tilde{M}}}_{v})(1+\delta_{v}g_{v}^{1}))/Q\right) \end{array}\right)\times (1-{\hat{A}}^{*}(\hat{\lambda }))\\ +{\hat{\lambda }}_{v}\left(E({\tilde{\tilde{𝕄}}}_{1b})+\omega_{2}E({\tilde{\tilde{𝕄}}}_{2b})+E({\tilde{\tilde{𝕄}}}_{r})\right)\times \\ \left(\begin{array}{c} \left({\tilde{\tilde{M}}}_{v}^{*}(Q)-1)X1(1-{\hat{A}}^{*}(\hat{\lambda })\right)\\ +(1-{\tilde{\tilde{M}}}_{v}^{*}(Q))-(1-{\tilde{\tilde{M}}}_{v}^{*}(Q))X1(-{\hat{\lambda }}_{v}{\overset{´}{b}}_{v}-{\hat{\lambda }}_{v}{\overset{´}{b}}_{v}\delta_{v}(g_{v}^{1})/Q) \end{array}\right)\times (1+\delta (g^{1}))\\ +\left(\begin{array}{c} {\hat{\lambda }}_{v}\left(\begin{array}{c} 1-X1(1-{\hat{A}}^{*}(\hat{\lambda }))-P1 \end{array}\right)(1-{\tilde{\tilde{𝕄}}}_{1b}^{*}(Q)/Q) \end{array}\right)\times (1+\delta_{v}(g_{v}^{1})) \end{array}\right\}}{\left(1-X1(1-{\hat{A}}^{*}(\hat{\lambda })-P1)\right)} \end{array}$$

## 5 Special cases

In this section, we investigate specific instances of our approach that correspond with contemporary research findings.

**Case (i)** No retrial, no phase-type service, and no re-service. Let $\dot{a}=\overset{´}{b}=p=0$; our analysis may seem streamlined to an *M*/*G*/1 with a working vacation queueing model. In this context, $K_{s}(\sigma )$ was derived as


$$\begin{array}{ccc} K_{s}(\sigma ) & = & \frac{\hat{\lambda }Q\left(1-\frac{\hat{\lambda }}{{\tilde{\tilde{𝕄}}}_{b}(\hat{\lambda }-\hat{\lambda }\sigma )}\right)[1-{\tilde{M}}_{v}(Q)]}{\left(Q(\hat{\lambda }+Q)+{\hat{\lambda }}^{2}(1-\sigma )(1-{\tilde{M}}_{v}(Q))-\hat{\lambda }\frac{\hat{\lambda }}{{\tilde{\tilde{𝕄}}}_{b}}Q(1-\sigma ){\tilde{M}}_{v}(Q)\right)}\times \\ & & \left\{\begin{array}{c} \frac{\left(\hat{\lambda }+Q)\sigma^{2}+(\hat{\lambda }(1-\sigma )+Q)[{\tilde{M}}_{v}(\hat{\lambda }+Q-\hat{\lambda }\sigma )-{\tilde{\tilde{M}}}_{b}(\hat{\lambda }-\hat{\lambda }\sigma )\right]}{\left[\sigma -{\tilde{\tilde{𝕄}}}_{b}(\hat{\lambda }-\hat{\lambda }\sigma )][\sigma -{\tilde{M}}_{v}(\hat{\lambda }+Q-\hat{\lambda }\sigma )\right]}\\ +\frac{\left(\begin{array}{c} \left(\begin{array}{c} (\hat{\lambda }+Q{)}^{2}\\ -{\hat{\lambda }}^{2}\sigma -\hat{\lambda }Q\sigma \end{array}\right){\tilde{\tilde{𝕄}}}_{b}(\hat{\lambda }-\hat{\lambda }\sigma )\frac{1}{\hat{\lambda }+Q-\hat{\lambda }\sigma }[1-{\tilde{M}}_{v}(\hat{\lambda }+Q-\hat{\lambda }\sigma )]\\ -\sigma [\hat{\lambda }(1+{\tilde{\tilde{𝕄}}}_{b}(\hat{\lambda }-\hat{\lambda }\sigma ))+Q(1+{\tilde{\tilde{𝕄}}}_{b}(\hat{\lambda }+Q-\hat{\lambda }\sigma ))] \end{array}\right)}{\left[\sigma -{\tilde{\tilde{𝕄}}}_{b}(\hat{\lambda }-\hat{\lambda }\sigma )][\sigma -{\tilde{M}}_{v}(\hat{\lambda }+Q-\hat{\lambda }\sigma )\right]} \end{array}\right\} \end{array}$$

This finding is consistent with the results obtained by Zhang and Hou [37].

**Case (ii)**: Without bulk arrival, no breakdown, no re-service. Let us define ${\hat{\lambda }}_{v}=\hat{\lambda }$, *p* = 0, $\delta_{v}=\delta$. The conceptual structure simplifies to an M/G/1 queueing model, which includes a balking customer and optional phase service under a vacation policy. The findings align with those reported by Arivudainambi and Godhandaraman [38]. In this case, we get


$$\begin{array}{ccc} & & K_{s}(\sigma )=\frac{U_{0}\begin{array}{c} \left(\begin{array}{c} (\sigma +(1-\sigma ){\hat{A}}^{*}(\hat{\lambda }))(1-{\tilde{\tilde{M}}}_{v}^{*}(\hat{\lambda }\overset{´}{b}(1-\sigma )))+(\overset{´}{b}-\sigma ){\hat{A}}^{*}(\hat{\lambda }){\tilde{\tilde{M}}}_{v}^{*}(\hat{\lambda }\overset{´}{b})\times U1\\ +(1-\overset{´}{b})\left(\begin{array}{c} \sigma {\hat{A}}^{*}(\hat{\lambda }){\tilde{\tilde{M}}}_{v}^{*}(\hat{\lambda }\overset{´}{b})-\sigma (1-{\hat{A}}^{*}(\hat{\lambda }))(1-{\tilde{\tilde{M}}}_{v}^{*}(\hat{\lambda }\overset{´}{b}(1-\sigma ))) \end{array}\right) \end{array}\right) \end{array}}{\overset{´}{b}{\tilde{\tilde{M}}}_{v}^{*}(\hat{\lambda }\overset{´}{b})\left\{(\sigma +(1-\sigma ){\hat{A}}^{*}(\hat{\lambda }))U1-\sigma \right\}} \end{array}$$

**Case (iii)** Without re-service, breakdowns, and bulk arrival. Assuming $\delta_{v}=p=0$, then the methodology reduces to a two-essential phase service with a working vacation incorporating an *M*/*G*/1 model. This coincides with the results obtained by Keerthiga and Indhira [39].


$$\begin{array}{cc} & K_{s}(\sigma )=\frac{U_{0}\left\{\begin{array}{c} \left(1+\frac{\hat{\lambda }(1-{\tilde{\tilde{M}}}_{v}^{*}(\delta +\hat{\lambda }\overset{´}{b})(1-\sigma ))}{\delta +\hat{\lambda }\overset{´}{b}(1-\sigma )}\right)\overset{´}{b}(\sigma )(\sigma -U2)\times \left(\begin{array}{c} {\hat{A}}^{*}\hat{\lambda }+(1+r-r\sigma )\\ \left(1-{\hat{A}}^{*}(\hat{\lambda })\right) \end{array}\right)\\ +\overset{´}{b}(\sigma )(1-{\hat{A}}^{*}\left(\hat{\lambda }\right))\sigma (U2A_{v}(\sigma )+{\tilde{\tilde{M}}}_{v}^{*}(A(\sigma ))-1)\\ +\hat{\lambda }\sigma \left\{\begin{array}{c} \sigma {\tilde{\tilde{M}}}_{v}(\sigma )+({\tilde{\tilde{M}}}_{v}^{*}(A(\sigma ))-1)({\hat{A}}^{*}\hat{\lambda }+(1+r-r\sigma )(1-{\hat{A}}^{*}(\hat{\lambda })))\times U2 \end{array}\right\} \end{array}\right\}}{\tau (\sigma )(\sigma -U2({\hat{A}}^{*}\hat{\lambda }+(1+r-r\sigma )(1-{\hat{A}}^{*}(\hat{\lambda }))))} \end{array}$$

## 6 Cost optimization

In this section, we analyze the cost structure associated with the proposed queueing model. A total cost function is formulated to evaluate system performance from an economic perspective and to determine an optimal service policy under different operational modes, including first-phase service (FPS), second-phase service (SPS), optional re-service, and working vacation (WV).

The expected total cost per unit time is obtained by combining the major cost components, namely holding cost, operating cost, setup cost, and idle cost. The total cost function is expressed as


$$\begin{array}{cc} TC & ={\mathbb{C}}_{h}L_{s}+{\tilde{C}}_{0}\frac{ℍ({𝕋}_{bs})}{ℍ({𝕋}_{bc})}+{\hat{C}}_{s}\frac{1}{ℍ({𝕋}_{bc})}+{\mathbb{C}}_{a}\frac{ℍ({𝕋}_{0})}{ℍ({𝕋}_{bc})}\\ & ={\mathbb{C}}_{h}L_{s}+{\tilde{C}}_{0}(1-U_{0})+{\hat{C}}_{s}\hat{\lambda }+{\mathbb{C}}_{a}U_{0}. \end{array}$$

Here, ${\mathbb{C}}_{h}$ is the holding cost per customer, ${\tilde{C}}_{0}$ is the server operating cost during a busy time, ${\hat{C}}_{s}$ is the setup cost for each service cycle, and ${\mathbb{C}}_{a}$ is the server idle cost. The term $L_{s}$ denotes the expected number of customers in the system, and *U*_0_ represents the server idle probability.

The cost components have clear operational interpretations. The holding cost corresponds to the customer waiting. The operational cost corresponds to the server utilization. The setup cost is charged for each service cycle and the idle cost corresponds to the cost of server inactivity.

For numerical illustration, we consider the parameter values $\hat{\lambda }={\hat{\lambda }}_{v}=1$, $\dot{a}=2$, $\beta_{1b}=6$, $\beta_{2b}=3$, $\beta_{r}=3$, $\beta_{v}=4$, *Q* = 2, $\eta_{1}=\eta_{2}=5$, $\eta_{r}=5$, and φ=3. Further, we assume $\delta =\delta_{v}=0.3$, *p* = 0.3, and $\overset{´}{b}=0.2$, along with cost parameters ${\mathbb{C}}_{h}=5$, ${\tilde{C}}_{0}=100$, ${\hat{C}}_{s}=500$, and ${\mathbb{C}}_{a}=90$. Under these settings, the expected total cost per unit time is obtained as *TC* = 440.4952.

**Fig 2:**
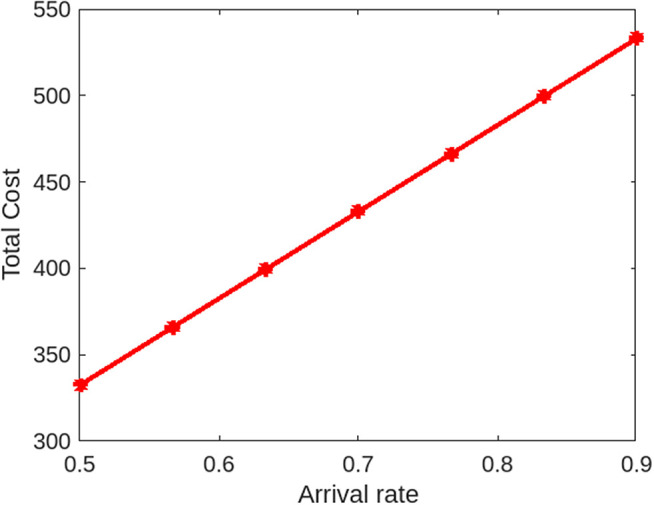
$TC\;Versus\;\hat{\lambda }$.

**Fig 3:**
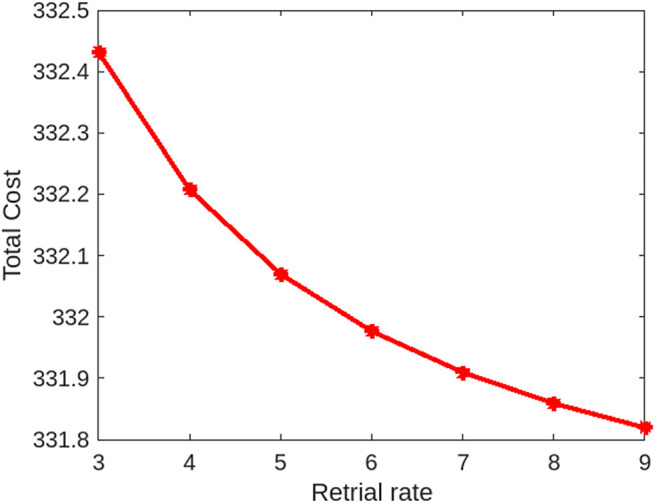
$TC\;Versus\;\dot{a}$.

**Fig 4:**
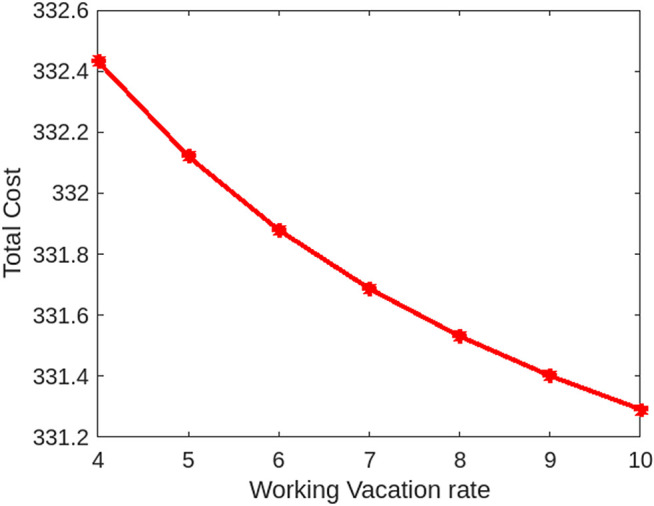
$TC\;Versus\;\beta_{v}$.

The results indicate that the total cost increases linearly with respect to the cost parameters, as observed in Tables 2–4. Sensitivity analysis is further performed by varying one parameter at a time while keeping others fixed. The corresponding numerical illustrations (Figs 2–4) demonstrate the impact of key system parameters such as the arrival rate ($\hat{\lambda }$), retrial rate ($\dot{a}$), and working vacation service rate ($\beta_{v}$) on the overall system cost.

**Table 2:** The impact of the parameters $\left({\mathbb{C}}_{h},\tilde{C_{0}}\right)$ on the anticipated cost function *TC* with $\hat{C_{s}}=$$600 and ${\mathbb{C}}_{a}=$$80.

(${\mathbb{C}}_{h},\tilde{C_{0}}$)	5, 100	5, 110	5, 120	10, 100	20, 100
*TC*	341.2397	342.4316	343.6235	341.2874	341.3829

**Table 3:** The impact of the parameters $\left(\tilde{C_{0}},{\mathbb{C}}_{a}\right)$ on the anticipated cost function *TC* with ${\mathbb{C}}_{h}=5$ and $\hat{C_{s}}=600$.

($\tilde{C_{0}},{\mathbb{C}}_{a}$)	100, 90	115, 90	125, 90	95, 100	105, 100
*TC*	341.2397	343.0275	344.2195	345.6437	354.4518

**Table 4:** The impact of the parameters $\left({\mathbb{C}}_{a},\hat{C_{s}}\right)$ on the anticipated cost function *TC* with ${\mathbb{C}}_{h}=$$5 and $\tilde{C_{0}}=$$80.

(${\mathbb{C}}_{a},\hat{C_{s}}$)	90, 500	95, 500	100, 500	90, 550	90, 600
*TC*	341.2397	345.6437	350.0477	366.2397	391.2397

It is worth noting that the suggested cost function is based on a linear combination of separate cost components for the purpose of analytical simplicity.This assumption makes the study simpler but may not reflect all aspects of actual world cost structures.

## 7 Numerical analysis and discussion

In this section, we analyze the impact of key system parameters on performance measures using numerical experiments. All stochastic processes, including retrial times, service times, re-service durations, and repair times, are assumed to follow exponential distributions for analytical tractability. Numerical results are obtained using a MATLAB program under stable operating conditions.

The probability density functions used in the analysis are given as follows. The exponential distribution is


$$\begin{array}{c} f(\overset{ˇ}{x})=\nu e^{-\nu \overset{ˇ}{x}},\;\overset{ˇ}{x}>0. \end{array}$$

The Erlang-2 distribution is defined as


$$\begin{array}{c} f(\overset{ˇ}{x})=\nu^{2}\overset{ˇ}{x}e^{-\nu \overset{ˇ}{x}},\;\overset{ˇ}{x}>0. \end{array}$$

The hyper-exponential distribution is given by


$$\begin{array}{c} f(\overset{ˇ}{x})=c\nu e^{-\nu \overset{ˇ}{x}}+(1-c)\nu^{2}e^{-\nu^{2}\overset{ˇ}{x}},\;\overset{ˇ}{x}>0. \end{array}$$

To illustrate system behavior, we evaluate key performance measures such as the server idle probability (*U*_0_), mean orbit size ($\pi_{q}$), and the probability of server unavailability during retrial (*T*).

For the selected metrics of $\hat{\lambda }\;\&\;{\hat{\lambda }}_{v}=1$; *Q* = 2; $\beta_{1b}=6;\beta_{2b}=6$; $beta_{r}=4$; *p* = 0.5; $\eta_{1}\;\&\;\eta_{2}=4$; φ=4; $\omega_{2}=0.2$; *X*1 = 1; $\overset{´}{b}\;\&\;\overset{´}{b_{v}}=0.2$ and $\beta_{v}=4$; $\delta \;\&\;\delta_{v}=0.3$.

### 7.1 Effect of retrial rate ($\dot{a}$)

From Table 5, it can be observed that as the retrial rate increases, the server idle probability *U*_0_ also increases, while the mean orbit size $\pi_{q}$ and server idle during retrial period *T* decreases. This happens because higher retrial attempts enable customers in the orbit to access the server more quickly, which in turn reduces congestion in the system.

**Table 5:** The effects of retrial customer rate $\dot{a}$ on $U_{0},\pi_{q},T$.

Retrial Rate $\dot{a}$	Exponential Distribution			Erlang Distribution			Hyper- Exponential		
	*U*_0_	$\pi_{q}$	*T*	*U*_0_	$\pi_{q}$	*T*	*U*_0_	$\pi_{q}$	*T*
3	0.7345	0.0255	0.0760	0.4327	0.0523	0.1460	0.8475	0.0145	0.0262
4	0.7567	0.0233	0.0627	0.4908	0.0496	0.1323	0.8562	0.0130	0.0196
5	0.7705	0.0220	0.0532	0.5281	0.0478	0.1209	0.8610	0.0121	0.0156
6	0.7800	0.0211	0.0461	0.5541	0.0466	0.1101	0.8640	0.0116	0.0130
7	0.7868	0.0205	0.0407	0.5732	0.0457	0.1006	0.8661	0.0112	0.0111

### 7.2 Effect of Failure Rate (δ)

From Table 6, it can be observed that as the breakdown rate increases, the server becomes unavailable more frequently, resulting in a decrease in the idle probability *U*_0_. The server idle time during the retrial period *T* also decreases. At the same time, the mean orbit size $\pi_{q}$ increases because more customers accumulate in the system during failure periods. This shows the real-world scenario of frequent failures leading to long waiting times and congestion.

**Table 6:** The effects of server breakdown rate δ on $U_{0},\pi_{q},T$.

breakdown rate δ	Exponential Distribution			Erlang Distribution			Hyper- Exponential		
	*U*_0_	$\pi_{q}$	*T*	*U*_0_	$\pi_{q}$	*T*	*U*_0_	$\pi_{q}$	*T*
0.1	0.7375	0.0287	0.0718	0.4500	0.1950	0.1442	0.8280	0.0151	0.0246
0.2	0.7360	0.0294	0.0716	0.4414	0.2145	0.1430	0.8276	0.0152	0.0245
0.3	0.7345	0.0302	0.0715	0.4327	0.2356	0.1418	0.8271	0.0153	0.0244
0.4	0.7330	0.0310	0.0714	0.4238	0.2584	0.1404	0.8267	0.0155	0.0243
0.5	0.7315	0.0319	0.0712	0.4148	0.2831	0.1389	0.8262	0.0156	0.0242

### 7.3 Effect of working vacation service rate ($\beta_{v}$)

From Table 7, it can be observed that as the service rate of the working vacation increases, the congestion in the system decreases, which leads to a reduction in the mean orbit size $\pi_{q}$ and also in the server idle time during retrial period *T*. At the same time, the server idle probability *U*_0_ increases, indicating better system performance even during the working vacation period.

**Table 7:** The effects of server on WV rate $\beta_{v}$ on $U_{0},\pi_{q},T$.

WV rate $\beta_{v}$	Exponential Distribution			Erlang Distribution			Hyper- Exponential		
	*U*_0_	$\pi_{q}$	*T*	*U*_0_	$\pi_{q}$	*T*	*U*_0_	$\pi_{q}$	*T*
2	0.6485	0.0489	0.0975	0.3610	0.2855	0.1597	0.6990	0.037	0.0440
3	0.6975	0.0375	0.0825	0.3983	0.2578	0.1503	0.7797	0.0221	0.0315
4	0.7345	0.0302	0.0715	0.4327	0.2356	0.1418	0.8271	0.0153	0.0244
5	0.7635	0.0252	0.0631	0.4638	0.2174	0.1340	0.8579	0.0116	0.0199
6	0.7868	0.0216	0.0564	0.4920	0.2021	0.1269	0.8794	0.0092	0.0168

### 7.4 Effect of balking rate ($\overset{´}{b}$)

The joining probability ($\overset{´}{b}$) increases, and the number of customers entering the system increases. Consequently, the server idle probability *U*_0_ decreases, while the orbit size $\pi_{q}$ and the probability that the server is idle during retrial time *T* increase due to uneven system flow and lower service consumption, as shown in Table 8.

**Table 8:** The effects of customer balking rate $\overset{´}{b}$ on $U_{0},\pi_{q},T$.

Balking rate $\overset{´}{b}$	Exponential Distribution			Erlang Distribution			Hyper- Exponential		
	*U*_0_	$\pi_{q}$	*T*	*U*_0_	$\pi_{q}$	*T*	*U*_0_	$\pi_{q}$	*T*
0.2	0.7465	0.0230	0.0482	0.4766	0.1807	0.1309	0.8307	0.0145	0.0218
0.3	0.7345	0.0272	0.0499	0.4327	0.2356	0.1404	0.8271	0.0155	0.0222
0.4	0.7219	0.0313	0.0516	0.3850	0.3227	0.1496	0.8234	0.0167	0.0225
0.5	0.7086	0.0354	0.0533	0.3333	0.4606	0.1588	0.8195	0.0180	0.0229
0.6	0.6946	0.0394	0.0550	0.2773	0.6879	0.1680	0.8155	0.0192	0.0232

### 7.5 Effect of re-service probability (*p*)

From Table 9, it can be observed that as the re-service probability *p* increases, more customers require additional service, which increases the system load. As a result, the mean orbit size $\pi_{q}$ increases, and the probability that the server is idle during the retrial period *T* also increases, and the server idle probability *U*_0_ decreases, indicating higher congestion in the system. The subsequent data visualizations indicate the effect of the features on system performance measures, thereby demonstrating the applicability of the findings to real-world scenarios.

**Table 9:** The effects of customers re-service rate *p* on $U_{0},\pi_{q},T$.

Re-service rate *p*	Exponential Distribution			Erlang Distribution			Hyper- Exponential		
	*U*_0_	$\pi_{q}$	*T*	*U*_0_	$\pi_{q}$	*T*	*U*_0_	$\pi_{q}$	*T*
0.20	0.7345	0.0302	0.0476	0.4327	0.2356	0.1276	0.8475	0.0153	0.0234
0.30	0.7319	0.0332	0.0478	0.4229	0.2685	0.1287	0.8469	0.0159	0.0236
0.40	0.7293	0.0364	0.0480	0.4130	0.3043	0.1298	0.8462	0.0165	0.0238
0.50	0.7267	0.0396	0.0482	0.4030	0.3433	0.1309	0.8456	0.0171	0.0241
0.60	0.7240	0.0429	0.0484	0.3927	0.3857	0.1320	0.8449	0.0177	0.0243

### 7.6 Graphical interpretation of results

In this part, we present a graphical analysis of the system performance measures, namely the mean orbit size $\left(\pi_{q}\right)$ and the server idle probability (*U*_0_). The surface and two-dimensional plots illustrate the relationship between system reliability, service efficiency, and retrial behavior.

#### 7.6.1 Effect on mean orbit size ($\pi_{q}$).

**Fig 5:**
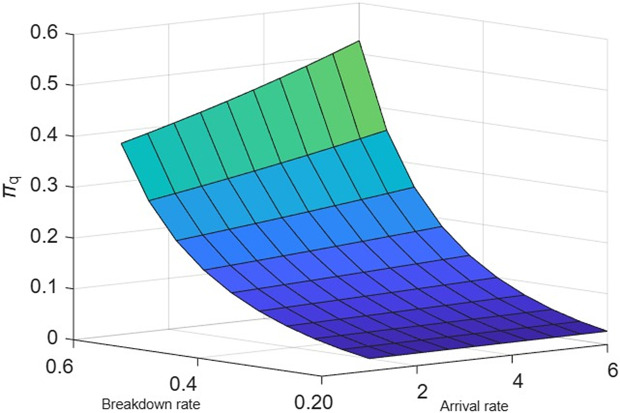
$\pi_{q}$ versus δ and $\hat{\lambda }$.

**Fig 6:**
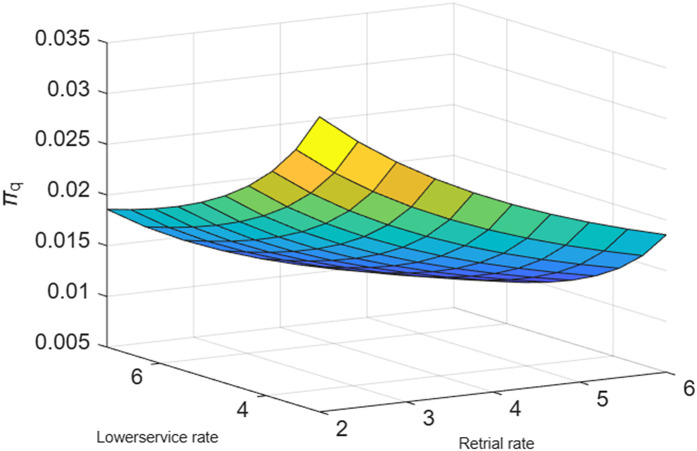
$\pi_{q}$ versus $\dot{a}$ and $\beta_{v}$.

**Fig 7:**
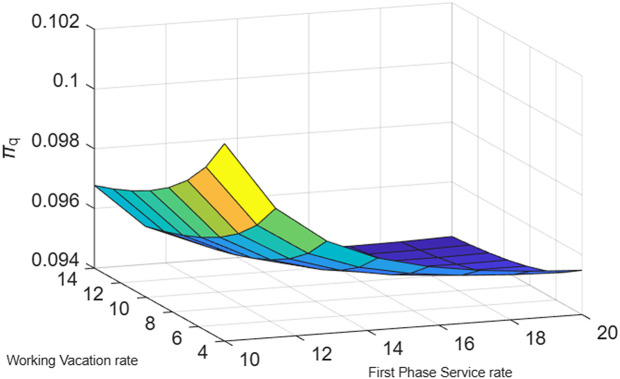
$\pi_{q}\;Versus\;\beta_{v}\;and\;\beta_{1b}$.

**Fig 8:**
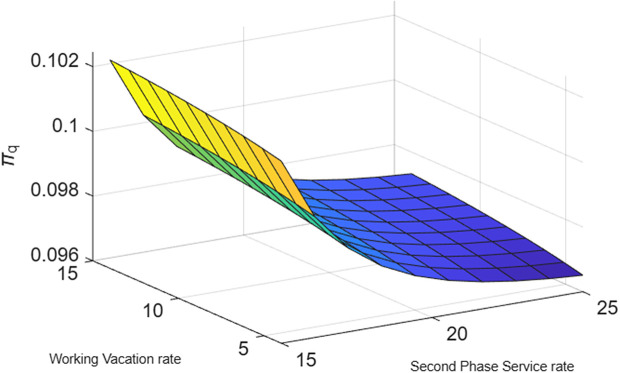
$\pi_{q}\;Versus\;\beta_{v}\;and\;\beta_{2b}$.

**Fig 9:**
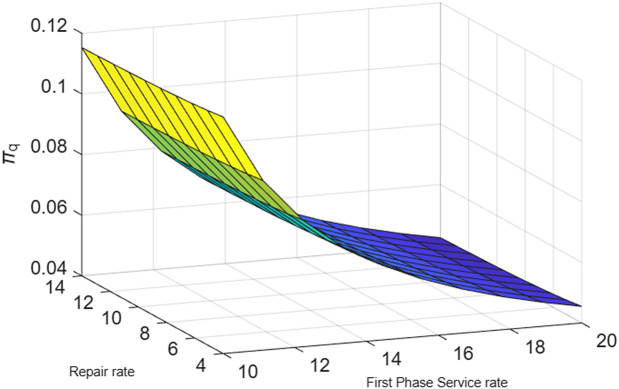
$\pi_{q}\;Versus\;\eta_{1}\;and\;\beta_{1b}$.

**Fig 10:**
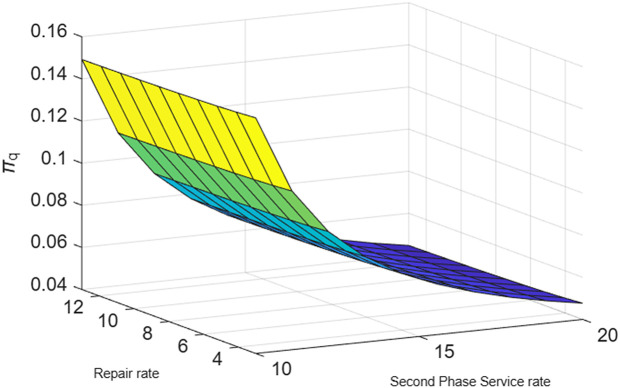
$\pi_{q}\;Versus\;\eta_{2}\;and\;\beta_{2b}$.

Fig 5-10 illustrate the variation of the mean orbit size ($\pi_{q}$) with respect to key system parameters.

- Fig 5 depicts the combined impact of arrival rate ($\hat{\lambda }$) and breakdown rate (δ). Larger arrival rates are often associated with increased congestion. However, under some situations, better service and repair efficiency might counteract this impact.
- Fig 6 indicates that the orbit size decreases significantly with the increase of the working vacation service rate ($\beta_{v}$) and retrial rate ($\dot{a}$) as the customers are more effectively serviced.
- Figs 7 and 8 show that the increased first-phase ($\beta_{1b}$) and second-phase service rates ($\beta_{2b}$) decrease $\pi_{q}$, which leads to more efficient service in many phases.
- Figs 9 and 10 show that as the repair rates ($\eta_{1}$ and $\eta_{2}$) increase, the server may be repaired faster and resume service sooner. Therefore, the system downtime is minimized. This leads to a reduction in mean orbit size ($\pi_{q}$).

#### 7.6.2 Effect on server idle probability (*U*_0_).

**Fig 11:**
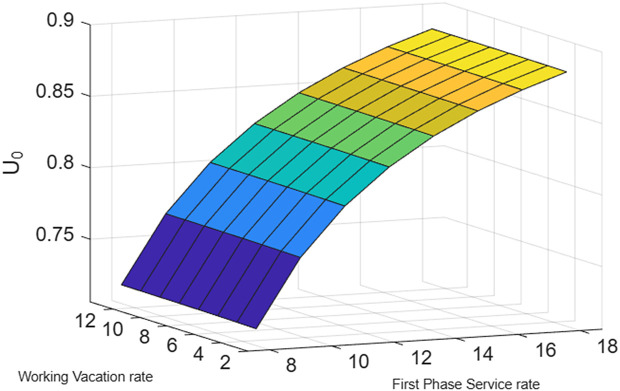
$U_{0}\;Versus\;\beta_{v}\;and\;\beta_{1b}$.

**Fig 12:**
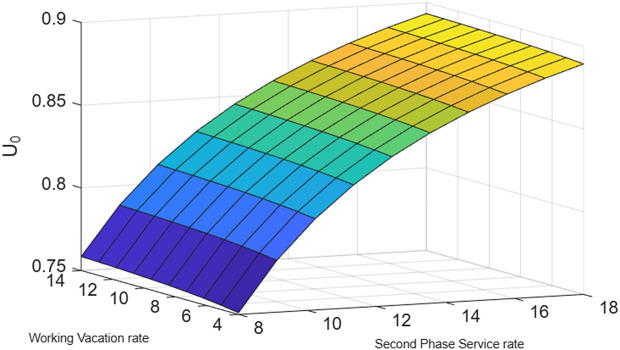
$U_{0}\;Versus\;\beta_{v}\;and\;\beta_{2b}$.

From Figs 11 and 12, it can be shown that the server idle probability (*U*_0_) is increasing with the larger working vacation service rate ($\beta_{v}$) and service rates ($\beta_{1b},\beta_{2b}$). This implies that shorter service completion times result in longer idle periods for the server.

**Fig 13:**
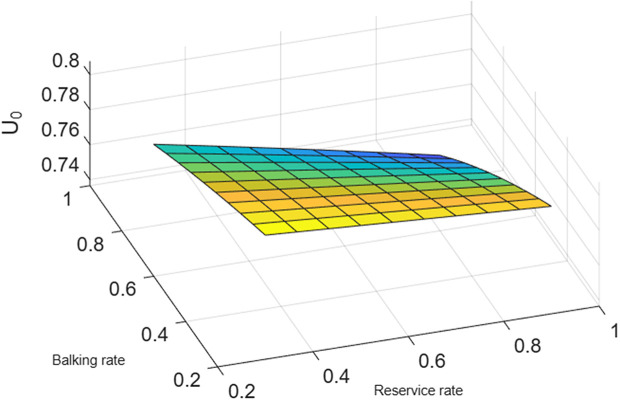
$U_{0}\;Versus\;\overset{´}{b}\;and\;p$.

Conversely, Fig 13 shows that the server idle probability (*U*0) decreases as the joining probability ($\overset{´}{b}$) and the re-service probability (*p*) increase, owing to the higher system workload.

#### 7.6.3 Combined parameter effects.

**Fig 14:**
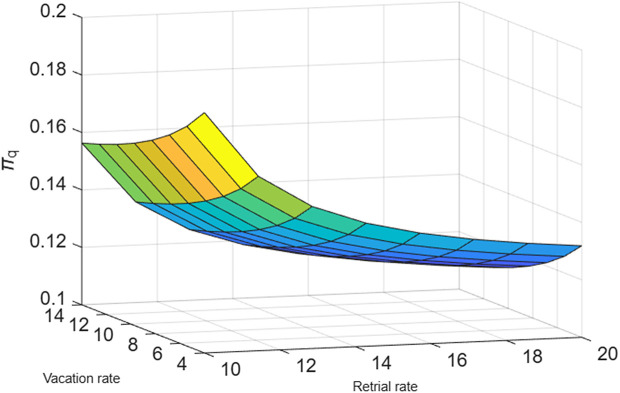
$\pi_{q}\;Versus\;Q\;and\;\dot{a}$.

Fig 14 shows the growth in the size of the orbit ($\pi_{q}$) with a combined increase in the vacation rate (*Q*) and retrial rate ($\dot{a}$) which suggests that high rate of retrials under high arrival rate could increase the congestion.

#### 7.6.4 Two-dimensional analysis.

**Fig 15:**
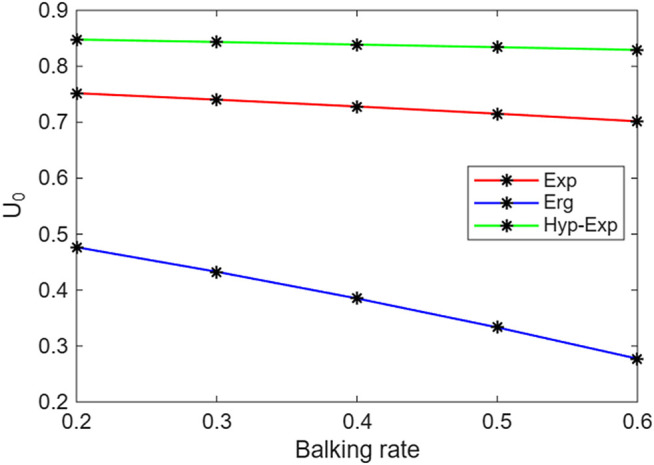
$U_{0}\;Versus\;\overset{´}{b}$.

**Fig 16:**
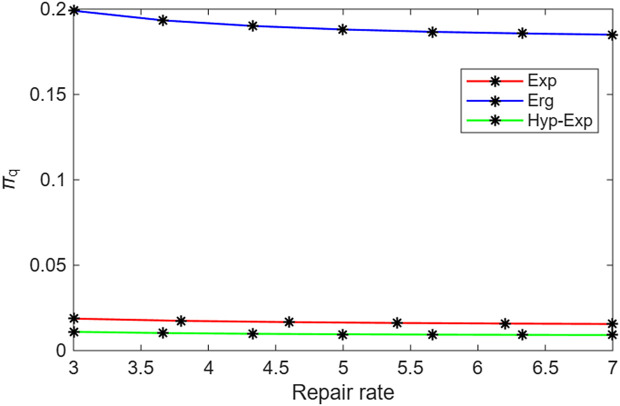
$\pi_{q}\;Versus\;\eta_{1}$.

**Fig 17:**
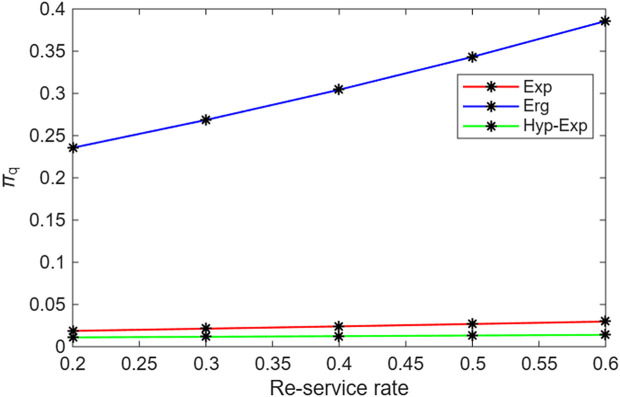
$\pi_{q}\;Versus\;p$.

**Fig 18:**
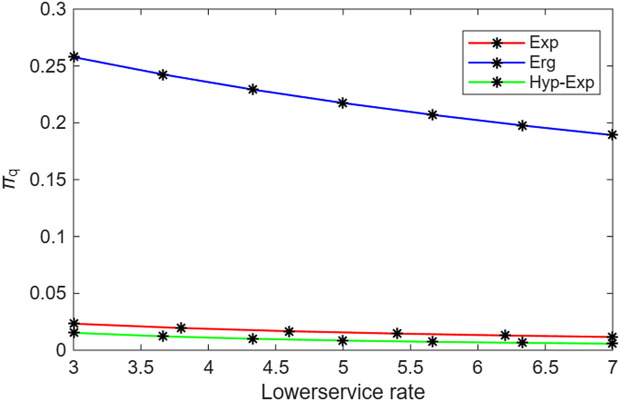
$\pi_{q}\;Versus\;\beta_{v}$.

Figs 15–18 further demonstrate the sensitivity of system performance:

- As shown in Fig 15, the server idle probability decreases with the growing balking rate, which is consistent with the shift of the effective system load.
- Fig 16 shows that the queue length ($\pi_{q}$) may decrease with an increase in the repair rate ($\eta_{1}$) due to the interaction between the repair and arrival processes.
- In certain cases, an increase in the re-service probability (*p*) leads to an increase in the orbit size $\pi_{q}$, indicating congestion caused by additional re-service requirements, as shown in Fig 17.
- As observed in Fig 18, an increase in the working vacation service rate ($\beta_{v}$) reduces the queue length of the system, indicating that improved service during working vacation helps stabilize system performance.

##### Managerial insights

The numerical and graphical data provide system administrators some crucial information. Higher service and repair rates substantially decrease congestion, emphasizing the necessity of effective resource allocation and maintenance planning in service systems. Higher retry rates also lower the size of the orbit. Thus, better customer retry mechanisms or system responsiveness may improve overall system performance.

On the other side, greater breakdown rates result in more congestion and worse system availability; hence, preventative maintenance measures are important. Similarly, an increased risk of re-service results in an increased system burden and points out the need for optimization of quality control procedures to prevent re-service requirements.

The impact of balking behavior implies that the impatience of customers may affect system utilization in many ways. Hence, the system efficiency may be improved by controlling customer expectations and minimizing waiting times.

Overall, our findings show that system performance is highly sensitive to service efficiency, system reliability, and retrial behavior, providing useful insights for the design and optimization of real-world production systems.

## 8 Conclusion

In this study, we analyzed a bulk arrival retrial queueing model incorporating customer balking, two-phase service, optional re-service, server breakdowns, and a working vacation policy. By applying the supplementary variable technique and probability generating functions, the system performance metrics such as the mean orbit size and server state probabilities were obtained.

A cost function was formulated to determine the optimal service rate under various operating conditions and to balance system performance and operating cost. Numerical and graphical analyses were performed to explore the effects of different system characteristics. Results demonstrate that the system performance is mostly dependent on service efficiency, retrial behavior, server reliability, and the re-service mechanism.

The proposed model is particularly applicable to multi-stage manufacturing systems, such as shoe production industries, where bulk arrivals, retrial behavior, rework, and machine breakdowns commonly occur. The obtained results provide useful insights for improving production planning, maintenance scheduling, and quality control in such environments.

Furthermore, the simultaneous integration of bulk arrivals, balking, retrial behavior, two-phase service, optional re-service, working vacation, and breakdown-repair mechanisms enables the proposed model to represent practical service and manufacturing systems more realistically than models that consider these features independently.

From a theoretical perspective, the proposed model generalizes several existing retrial queueing models as special cases, thereby extending earlier results and contributing to the development of retrial queueing theory.

### 8.1 Limitations and future research

In the present study, the main system parameters, such as the balking probability, retrial rate, and service rates, are assumed to be fixed constants chosen to satisfy the system stability condition. However, in practical service systems, these factors may change constantly depending on system congestion, customer behavior, and pricing schemes.

In particular, the balking customers may depend on factors including queue size, waiting time, and service cost, leading to state-dependent or adaptive balking probability. Similarly, retrial attempts and service capacities may be dynamically adjusted to improve overall system performance.

Another limitation of the present study is the formulation of the total cost function. For convenience in analysis, the proposed cost model is based on a linear combination of individual cost components. This assumption is useful in simplifying the analysis and optimization but may not fully illustrate the actual cost structure in real-world systems.

In practice, the costs associated with extended waiting periods, extreme congestion, or repeated retrials are generally non-linear. For instance, the penalty charges or the customer dissatisfaction may increase rapidly when the waiting time exceeds an acceptable limit. Therefore, the inclusion of non-linear or congestion-dependent cost functions would provide a more precise illustration of the system economics.

The proposed model can be extended in future work by including dynamic control mechanisms such as congestion-dependent balking, adaptive retrial policies, dynamic resource allocation, and non-linear cost functions. These extensions would enhance the practical applicability of the model but also contribute to the analytical complexity of the model.

## Supporting information

S1 Appendix The calculations for the average orbit size and average system size are provided in this appendix. (PDF)
