# Supplementary material for: Reliability and performance modeling of an M[X]/G(a,b)/1 retrial queue with re-service and Balking behavior under working vacation policy
Source: PLoS One. 2026 Aug 6;21(8):e0354900. doi: 10.1371/journal.pone.0354900 (PMC13446710; doi:10.1371/journal.pone.0354900)
Supplement: Supplementary file 1 [file pone.0354900.s001.pdf]

# S1 Appendix

$$
Nu_o^{''}(1) = -2 \left\{ \begin{array}{r} \hat{\lambda}_v \left(P1 - pE(\tilde{\tilde{\mathbb{M}}}_r)\hat{\lambda}\acute{b}X1(1+\delta g^{1r})(1+w2)\right) \begin{pmatrix} (\tilde{\tilde{M}}_v^*(Q)-1)X1(1-\hat{\mathbb{A}}^*(\hat{\lambda})) + (1-\tilde{\tilde{M}}_v^*(Q)) \\ + \left((1-\tilde{\tilde{M}}_v^*(Q))(\hat{\lambda}_v\acute{b}_vX1\tilde{\tilde{M}}_v^*(Q)(1+\delta_v g_v^1))/Q\right) \end{pmatrix} \\ +\hat{\lambda}\acute{b}(1-\hat{\mathbb{A}}^*(\hat{\lambda}))X1 \left(P1(1-\tilde{\tilde{M}}_v^*(Q)) + \dfrac{(1-\tilde{\tilde{M}}_v^*(Q))}{Q}\hat{\lambda}_v\acute{b}_v\tilde{\tilde{M}}_v(1+\delta_v g_v^{(1)})\right) \\ + \left(1 - X1(1-\hat{\mathbb{A}}^*(\hat{\lambda})) - P1\right) \times \left(\hat{\lambda}\acute{b}X1 + \hat{\lambda}_v^2\acute{b}_v^2\tilde{\tilde{M}}_v^*(Q)\delta_v\dfrac{(1-\tilde{\tilde{M}}_v^*(Q))}{Q} + \hat{\lambda}\acute{b}\hat{\lambda}_v\acute{b}_v\dfrac{(1-\tilde{\tilde{M}}_v^*(Q))}{Q}\right) \end{array} \right\}
$$

$$
\begin{aligned}
&Nu_o^{'''}(1) = \\
&\left(-3\hat{\lambda}\acute{b}X1 - 3\frac{\hat{\lambda}\acute{b}X1(1-\tilde{\tilde{M}}_v^*(Q)}{Q} - 3\delta_v\frac{\hat{\lambda}_v^2 g_v\acute{b}_v^2X1(1-\tilde{\tilde{M}}_v^*(Q))}{Q}\right) \times \\
&\begin{pmatrix} -2X1(1-\hat{\mathbb{A}}^*(\hat{\lambda}))P1 - E(\tilde{\tilde{\mathbb{M}}}_{1b})^2\hat{\lambda}^2\acute{b}^2X1^2(-1-\delta g^{1f})^2 - E(\tilde{\tilde{\mathbb{M}}}_{1b})\hat{\lambda}^2\acute{b}^2X1^2(\delta g^{2f}) \\ -2E(\tilde{\tilde{\mathbb{M}}}_{1b})^2\hat{\lambda}^2\acute{b}^2X1^2(-1-\delta g^{1f})^2w2E(\tilde{\tilde{\mathbb{M}}}_{2b}) - 2E(\tilde{\tilde{\mathbb{M}}}_{1b})\hat{\lambda}\acute{b}X1(1+\delta g^1)pE(\tilde{\tilde{\mathbb{M}}}_r)\hat{\lambda}\acute{b}X1(1+\delta g^{1r}) \\ -w2E(\tilde{\tilde{\mathbb{M}}}_{2b})^2\hat{\lambda}^2\acute{b}^2X1^2(-1-\delta g^{1s})^2 - w2E(\tilde{\tilde{\mathbb{M}}}_{2b})\hat{\lambda}^2\acute{b}^2X1^2(\delta g^{2s}) \\ -2w2E(\tilde{\tilde{\mathbb{M}}}_{2b})\hat{\lambda}\acute{b}X1(1+\delta g^{1s})pE(\tilde{\tilde{\mathbb{M}}}_r)\hat{\lambda}\acute{b}X1(1+\delta g^{1r}) \\ -pE(\tilde{\tilde{\mathbb{M}}}_r)^2\hat{\lambda}^2\acute{b}^2X1^2(-1-\delta g^{1r})^2 - pE(\tilde{\tilde{\mathbb{M}}}_r)\hat{\lambda}^2\acute{b}^2X1^2(\delta g^{2r}) \end{pmatrix}
\end{aligned}
$$

$$
\begin{aligned}
&-3\hat{\lambda}_v \begin{pmatrix} pE(\tilde{\tilde{\mathbb{M}}}_r)^2\hat{\lambda}^2\acute{b}^2X1(-1-\delta g^{1r})^2(1+w2) + 3\hat{\lambda}_v pE(\tilde{\tilde{\mathbb{M}}}_r)^2\hat{\lambda}^2\acute{b}^2X1(-\delta g^{2r})(1+w2) \\ -6\hat{\lambda}bX1\left(-E(\tilde{\tilde{\mathbb{M}}}_{1b})(1+\delta g^{1f}) - w2E(\tilde{\tilde{\mathbb{M}}}_{1b})(1+\delta g^{1f})\right) \end{pmatrix} \times \\
&\left((\tilde{\tilde{M}}_v^*(Q)-1)X1(1-\hat{\mathbb{A}}^*(\hat{\lambda})) + (1-\tilde{\tilde{M}}_v^*(Q)) + \left((1-\tilde{\tilde{M}}_v^*(Q))(\hat{\lambda}_v\acute{b}_vX1\tilde{\tilde{M}}_v^*(Q)(1+\delta_v g_v^1))/Q\right)\right) \\
&-6\hat{\lambda}\acute{b}(1-\hat{\mathbb{A}}^*(\hat{\lambda}))\left(\left(P1(1-\tilde{\tilde{M}}_v^*(Q))\right) + \left((1-\tilde{\tilde{M}}_v^*(Q))(\hat{\lambda}_v\tilde{\tilde{M}}_v^*(1+\delta_v g_v^1))/Q\right)\right) \\
&-3\hat{\lambda}bX1(1-\hat{\mathbb{A}}^*(\hat{\lambda})) \begin{pmatrix} 2\left(P1(1-\tilde{\tilde{M}}_v^*(Q))\right)V^{'}(1) + V^{''}(1) + (1-\tilde{\tilde{M}}_v^{*'}(Q))P2 + (1-\tilde{\tilde{M}}_v^{*'}(Q))P3 \\ 2(1-\tilde{\tilde{M}}_v^{*'}(Q))E(\tilde{\tilde{\mathbb{M}}}_{1b})\hat{\lambda}\acute{b}X1(1+\delta_v g_v^1)\begin{pmatrix} w2E(\tilde{\tilde{\mathbb{M}}}_{2b})\hat{\lambda}\acute{b}X1(1+\delta_v g^{1s}) \\ +pE(\tilde{\tilde{\mathbb{M}}}_r)\hat{\lambda}\acute{b}X1(1+\delta_v g^{1r}) \end{pmatrix} \\ +(\tilde{\tilde{M}}_v^{*''}(Q))\left(\hat{\lambda}_v\acute{b}_v\tilde{\tilde{M}}_v^*(Q)(1+\delta_v g_v^1)\right)^2 - (\tilde{\tilde{M}}_v^{*'}(Q))\hat{\lambda}_v^2\acute{b}_v^2\delta_v g_v^2 \end{pmatrix} \\
&+\frac{1}{Q}\left(1 - X1(1-\hat{\mathbb{A}}^*(\hat{\lambda})) - P1\right) \times \left(-6\hat{\lambda}_v V^{'}(1)g_v^1\hat{\lambda}_v^2\acute{b}_v^2\tilde{\tilde{M}}_v^*(Q) - 3\delta_v V(1)g_v^2\hat{\lambda}_v^3\acute{b}_v^3\tilde{\tilde{M}}_v(Q)^{*2} - 6\hat{\lambda}\acute{b}\hat{\lambda}_v\acute{b}_v\delta_v V^{'}(1)\right) \\
&-6\hat{\lambda}_v\acute{b}_v\delta_v p\tilde{\tilde{M}}_r^*\hat{\lambda}\acute{b}X1(1+\delta g^{1r}) \begin{pmatrix} E(\tilde{\tilde{\mathbb{M}}}_{1b})^2\hat{\lambda}\acute{b}X1(1+\delta g^{1f}) \\ +w2E(\tilde{\tilde{\mathbb{M}}}_{1b})^2\hat{\lambda}\acute{b}X1(1+\delta g^{1s}) \\ +w2E(\tilde{\tilde{\mathbb{M}}}_{2b})^2\hat{\lambda}\acute{b}X1(1+\delta g^{2s}) \end{pmatrix} \begin{pmatrix} (\tilde{\tilde{M}}_v^*(Q)-1)X1(1-\hat{\mathbb{A}}^*(\hat{\lambda})) + (1-\tilde{\tilde{M}}_v^*(Q)) \\ + \left((1-\tilde{\tilde{M}}_v^*(Q))(\hat{\lambda}_v\tilde{\tilde{M}}_v(Q)(1+\delta_v g_v^1))/Q\right) \end{pmatrix} \\
&-3\hat{\lambda}_v p\tilde{\tilde{M}}_r^*\hat{\lambda}\acute{b}X1(1+\delta g^{1r})(1+w2) \begin{pmatrix} (\tilde{\tilde{M}}_v^*)^{*''}(Q)\hat{\lambda}_v^2\acute{b}_v^2X1(-1-\delta g_v^1)^2 + (\tilde{\tilde{M}}_v^{*'})(Q)\hat{\lambda}_v^2\acute{b}_v^2X1^2(-\delta g_v^2) \\ +2E(\tilde{\tilde{M}}_v^{*'}(Q))\hat{\lambda}_v\acute{b}_vX1(-1-\delta g_v^1)X1(1-\hat{\mathbb{A}}) + 2V^{'}(1) + V^{''}(1) \end{pmatrix}
\end{aligned}
$$

$$-3\hat{\lambda}_v \begin{pmatrix} E(\tilde{\tilde{M}}_{1b})^2(\hat{\lambda}\acute{b}(-1-\delta g^{1f}))^2+ \\ \omega_2 E(\tilde{\tilde{M}}_{2b})(\hat{\lambda}\acute{b}(-1-\delta g^{1s})) \end{pmatrix} \begin{pmatrix} (\tilde{\tilde{M}}_v^{*''})(Q)\hat{\lambda}_v^2\acute{b}_v^2 X1(-1-\delta g_v^1)^2 + (\tilde{\tilde{M}}_v)^{*'}(Q)\hat{\lambda}_v^2\acute{b}_v^2 X1^2(-\delta g_v^2) \\ +2E(\tilde{\tilde{M}}_v^{*'}(Q))\hat{\lambda}_v\acute{b}_v X1(-1-\delta g_v^1)X1(1-\hat{\mathbb{A}})+2V'(1)+V''(1) \end{pmatrix}$$

$$\begin{aligned}
De_s^{''}(1) &= -2\hat{\lambda}X1\acute{b}\left(1-X1(1-\hat{\mathbb{A}}^*(\hat{\lambda}))-P1\right) \\
De_s^{'''}(1) &= -3\hat{\lambda}\acute{b}X1 \begin{pmatrix} -2X1(1-\hat{\mathbb{A}}^*(\hat{\lambda}))P1 \\ -E(\tilde{\tilde{\mathbb{M}}}_{1b})^2\hat{\lambda}^2\acute{b}^2X1^2(-1-\delta g^{1f})^2 - E(\tilde{\tilde{\mathbb{M}}}_{1b})\hat{\lambda}^2\acute{b}^2X1^2(\delta g^{2f}) \\ -2E(\tilde{\tilde{\mathbb{M}}}_{1b})^2\hat{\lambda}^2\acute{b}^2X1^2(-1-\delta g^{1f})^2 w2E(\tilde{\tilde{\mathbb{M}}}_{2b}) \\ -2E(\tilde{\tilde{\mathbb{M}}}_{1b})\hat{\lambda}\acute{b}X1(1+\delta g^{1f})pE(\tilde{\tilde{\mathbb{M}}}_r)\hat{\lambda}\acute{b}X1(1+\delta g^{1r}) \\ -w2E(\tilde{\tilde{\mathbb{M}}}_{2b})^2\hat{\lambda}^2\acute{b}^2X1^2(-1-\delta g^{2s})^2 - w2E(\tilde{\tilde{\mathbb{M}}}_{2b})\hat{\lambda}^2\acute{b}^2X1^2(\delta g^{1s}) \\ -2w2E(\tilde{\tilde{\mathbb{M}}}_{2b})\hat{\lambda}\acute{b}X1(1+\delta g^{1s})pE(\tilde{\tilde{\mathbb{M}}}_r)\hat{\lambda}\acute{b}X1(1+\delta g^{1r}) \\ -pE(\tilde{\tilde{\mathbb{M}}}_r)^2\hat{\lambda}^2\acute{b}^2X1^2(-1-\delta g^{2r})^2 - pE(\tilde{\tilde{\mathbb{M}}}_r)\hat{\lambda}^2\acute{b}^2X1^2(\delta g^{2r}) \end{pmatrix} \\
Nu_s^{'''}(1) &= Nu_0^{'''}(1) - 3\hat{\lambda}\acute{b}X1(1-\hat{\mathbb{A}}^*(\hat{\lambda}))\left(P1(1-\tilde{\tilde{M}}_v^*(Q)) + (1-\tilde{\tilde{M}}_v^*(Q))/Q\right) \\
&\quad -6\hat{\lambda}_v\tilde{\tilde{M}}_v P1\left(\tilde{\tilde{M}}_v^*(Q) + (\tilde{\tilde{M}}_v^*(Q)-1)X1(1-\hat{\mathbb{A}}^*(\hat{\lambda})) + (1-\tilde{\tilde{M}}_v^*(Q)) + \frac{\tilde{\tilde{M}}_v^*(Q)}{Q}\right)
\end{aligned}$$

Where,

$$\begin{aligned}
&A_{1b}(\sigma) = (\hat{\lambda}\acute{b}(1-X(\sigma)) + \delta(1-W_{1b}^*(\tau(\sigma)))) \\
&A_{2b}(\sigma) = (\hat{\lambda}\acute{b}(1-X(\sigma)) + \delta(1-W_{2b}^*(\tau(\sigma)))) \\
&A_r(\sigma) = (\hat{\lambda}\acute{b}(1-X(\sigma)) + \delta(1-W_r^*(\tau(\sigma)))) \\
&A_v(\sigma) = (\hat{\lambda}_v(1-X(\sigma)) + Q + \delta_v(1-G_w^*(\tau_v(\sigma)))) \\
&\tau(\sigma) = \hat{\lambda}\acute{b}(1-X(\sigma)) \\
&\tau_v(\sigma) = \hat{\lambda}_v\acute{b}_v(1-X(\sigma)) \\
&V(\sigma) = \frac{Q\ \left[1-\tilde{\tilde{M}}_v^*\left(A_v(\sigma)\right)\right]}{A_v(\sigma)}
\end{aligned}$$

$$\begin{aligned}
P1 &= \left(E(\tilde{\tilde{\mathbb{M}}}_{1b})(\hat{\lambda}\acute{b}X1(1+\delta g^{1f})) + \omega_2E(\tilde{\tilde{\mathbb{M}}}_{2b})(\hat{\lambda}\acute{b}X1(1+\delta g^{1s})) + E(\tilde{\tilde{\mathbb{M}}}_r)(\hat{\lambda}\acute{b}X1(1+\delta g^{1r}))\right) \\
P2 &= \left(E(\tilde{\tilde{\mathbb{M}}}_{1b})^2(\hat{\lambda}^2\acute{b}^2X1^2(1+\delta g^{1f}))^2 + \omega_2E(\tilde{\tilde{\mathbb{M}}}_{2b})^2(\hat{\lambda}^2\acute{b}^2X1^2(1+\delta g^{1s}))^2 + pE(\tilde{\tilde{\mathbb{M}}}_r)^2(\hat{\lambda}^2\acute{b}^2X1^2(1+\delta g^{1r}))^2\right) \\
P3 &= \left(E(\tilde{\tilde{\mathbb{M}}}_{1b})\hat{\lambda}^2\acute{b}^2X1^2(\delta g^{1f}) + E(\tilde{\tilde{\mathbb{M}}}_{2b})\hat{\lambda}^2\acute{b}^2X1^2(\delta g^{1s}) + E(\tilde{\tilde{M}}_r\hat{\lambda}^2\acute{b}^2X1^2(\delta g^{1r}))\right)
\end{aligned}$$

$$\begin{aligned}
U1 &= (\omega_1 + \omega_2\tilde{\tilde{\mathbb{M}}}_{2b}^*(\hat{\lambda}\acute{b}X1(1-\sigma)))\tilde{\tilde{\mathbb{M}}}_{1b}^*(\hat{\lambda}\acute{b}X1(1-\sigma)) \\
U2 &= \omega_1\tilde{\tilde{\mathbb{M}}}_{1b}^*(A_{1b}(\sigma)) + \omega_2\tilde{\tilde{\mathbb{M}}}_{1b}^*(A_{1b}(\sigma))\tilde{\tilde{\mathbb{M}}}_{2b}^*(A_{2b}(\sigma)) \\
\rho &= X1(1-\hat{\mathbb{A}}^*(\hat{\lambda})) + P1
\end{aligned}$$
